# RopB protein of *Rhizobium leguminosarum* bv. *viciae* adopts amyloid state during symbiotic interactions with pea (*Pisum sativum* L.)

**DOI:** 10.3389/fpls.2022.1014699

**Published:** 2022-10-24

**Authors:** Anastasiia O. Kosolapova, Mikhail V. Belousov, Maksim I. Sulatsky, Anna V. Tsyganova, Anna I. Sulatskaya, Alexander G. Bobylev, Oksana Y. Shtark, Viktor E. Tsyganov, Kirill V. Volkov, Vladimir A. Zhukov, Igor A. Tikhonovich, Anton A. Nizhnikov

**Affiliations:** ^1^ All-Russia Research Institute for Agricultural Microbiology, St. Petersburg, Russia; ^2^ St. Petersburg State University, St. Petersburg, Russia; ^3^ Institute of Cytology, Russian Academy of Sciences, St. Petersburg, Russia; ^4^ Institute of Theoretical and Experimental Biophysics, Russian Academy of Sciences, Pushchino, Russia

**Keywords:** RopB, amyloid, nodule, plant-microbe interactions, symbiosis, outer membrane protein, *Pisum sativum* L., beta-barrel

## Abstract

Amyloids represent protein aggregates with highly ordered fibrillar structure associated with the development of various disorders in humans and animals and involved in implementation of different vital functions in all three domains of life. In prokaryotes, amyloids perform a wide repertoire of functions mostly attributed to their interactions with other organisms including interspecies interactions within bacterial communities and host-pathogen interactions. Recently, we demonstrated that free-living cells of *Rhizobium leguminosarum*, a nitrogen-fixing symbiont of legumes, produce RopA and RopB which form amyloid fibrils at cell surface during the stationary growth phase thus connecting amyloid formation and host-symbiont interactions. Here we focused on a more detailed analysis of the RopB amyloid state *in vitro* and *in vivo*, during the symbiotic interaction between *R. leguminosarum* bv. *viciae* with its macrosymbiont, garden pea (*Pisum sativum* L.). We confirmed that RopB is the *bona fide* amyloid protein since its fibrils exhibit circular x-ray reflections indicating its cross-β structure specific for amyloids. We found that fibrils containing RopB and exhibiting amyloid properties are formed *in vivo* at the surface of bacteroids of *R. leguminosarum* extracted from pea nodules. Moreover, using pea *sym31* mutant we demonstrated that formation of extracellular RopB amyloid state occurs at different stages of bacteroid development but is enhanced in juvenile symbiosomes. Proteomic screening of potentially amyloidogenic proteins in the nodules revealed the presence of detergent-resistant aggregates of different plant and bacterial proteins including pea amyloid vicilin. We demonstrated that preformed vicilin amyloids can cross-seed RopB amyloid formation suggesting for probable interaction between bacterial and plant amyloidogenic proteins in the nodules. Taken together, we demonstrate that *R. leguminosarum* bacteroids produce extracellular RopB amyloids in pea nodules *in vivo* and these nodules also contain aggregates of pea vicilin amyloid protein, which is able to cross-seed RopB fibrillogenesis *in vitro.* Thus, we hypothesize that plant nodules contain a complex amyloid network consisting of plant and bacterial amyloids and probably modulating host-symbiont interactions.

## 1 Introduction

Amyloids are highly ordered unbranched fibrillar protein aggregates that have been proven to play a part not only in the development of the incurable diseases of humans and animals but also perform biological functions (Sawaya et al., 2021). Specific spatial structure of amyloids called “cross-β” (Eanes and Glenner, 1968) gives them unique properties including resistance to treatment with ionic detergents, binding with Congo red (CR) and Thioflavin T (ThT) dyes, and cross-β diffraction pattern at x-ray diffraction analysis (Nizhnikov et al., 2015). To date, more than 30 functional amyloids of prokaryotes, part of which are involved in interspecies interactions, including interactions within microbial communities, host-pathogen, and host-symbiont interactions, have been identified (Kosolapova et al., 2020). One group of amyloidogenic proteins involved in interspecies interactions are outer membrane proteins (OMPs). OMPs are β-barrel proteins that are found within Gram-negative bacteria, chloroplasts, and mitochondria (Otzen and Andersen, 2013). Several OMPs of *Proteobacteria*, including OmpA and OmpC proteins of *Escherichia coli* (Danoff and Fleming, 2015; Joseph Sahaya Rajan et al., 2016), OmpP2-like protein of *Mannheimia haemolytica* (Montes García et al., 2018), RopA and RopB proteins of *Rhizobium leguminosarum* (Kosolapova et al., 2019), can form amyloid or amyloid-like fibrils and are hypothesized to be involved in bacterial virulence and promote adhesion in their amyloid form (Kosolapova et al., 2020).

RopB is a protein of *R. leguminosarum* that has a predicted structure of β-sheet-rich OmpA-like outer membrane β-barrel (Roest et al., 1995). The *R. leguminosarum* bacterium belongs to the order *Rhizobiales*, class *Alphaproteobacteria* and as the other rhizobia is able to form nodules on the roots of legumes (Young et al., 2021). In symbiotic nodules, rhizobial cells differentiate to bacteroids that form organelle-like symbiosomes, in which nitrogen fixation takes place (Tsyganova et al., 2019). Garden pea (*Pisum sativum* L.) is a specific symbiotic partner of *R. leguminosarum* bv. *viciae* (Laguerre et al., 2007), for which the extensive collections of symbiotic mutants with various abnormalities in the nodule development were obtained (Tsyganov and Tsyganova, 2020). In particular, Sprint-2Fix^–^ (*sym31*) mutant is characterized by formation of multibacteroid symbiosomes filled with undifferentiated bacteroids (Borisov et al., 1992; 1994). The juvenile state of bacteroids and symbiosomes in the *sym31* mutant was clearly demonstrated by several parameters. Firstly, colony-forming units from the *sym31* mutant nodules are more abundant than those of wild type and mutants blocked at later stages of nodule development (Tsyganov et al., 2003). The vesicle targeting pathway implicated in symbiosome development is abnormal in the *sym31* mutant whichleads to the accumulation of the PsNLEC-1 glycoprotein in the vacuole in mutant nodules instead of symbiosomes (Dahiya et al., 1998). The symbiosome membranes in the *sym31* mutant lack the arabinogalactan protein, recognized by the JIM1 antibody, which is abundantly present in mature symbiosomes in wild-type nodules (Tsyganova et al., 2019). The other glycoprotein, recognized by the MAC 266 antibody, which is accumulated in mature symbiosomes in the wild-type nodules, was present only in rare mature cells in the *sym31* mutant (Sherrier et al., 1997). The presence of glycolipid antigen, recognized by the antibody JIM18, in all symbiosome membranes in the *sym31* mutant also confirms their juvenile state, since this antigen is present only in juvenile symbiosome membranes in wild-type nodules (Sherrier et al., 1997). It is important to note that there is no leghemoglobin in the nodules of the *sym31* mutant, the activity of nitrogen assimilation enzymes is altered, and the content of ononitol is reduced (Romanov et al., 1995). In addition, the *sym31* mutant manifests nitrate tolerance (Rosov et al., 2001). Thus, the *sym31* mutant is a convenient model for studying bacteroid differentiation.

The precise function of the RopB protein of *R. leguminosarum* remains unknown. It is hypothesized that RopB plays a role in outer membrane stability as *ropB* mutants demonstrate increased sensitivity to detergents and organic acids (Foreman et al., 2010). The increase in expression of RopB has been reported at the early stages of nodulation (Tolin et al., 2013). At the same time, the expression of *RopB* is decreased dramatically in nodules compared to free-living *R. leguminosarum* culture (Foreman et al., 2010). RopB has also been reported to be absent in the outer membrane of bacteroids (Roest, 1995). In contrast, Wheatley et al. classified *ropB* as a “rhizosphere-progressive gene”, i.e., a gene required across all symbiosis establishment stages, using mariner-based transposon insertion sequencing (INSeq) (Wheatley et al., 2020). Thus, despite some contradictions the data obtained suggest that RopB can be involved in the control of symbiont-host interactions.

Previously, we have reported that RopB forms amyloid fibrils *in vitro* (Kosolapova et al., 2019). What is more, we have found that during the late stationary phase some fraction of RopB is localized in fibrous CR binding capsule of the free-living *R. leguminosarum* cells that exhibits green birefringence, which is an argument for the *in vivo* amyloid formation by RopB (Kosolapova et al., 2019).

In this work, we continued to analyze the amyloid properties and structural characteristics of RopB protein with the usage of x-ray diffraction analysis and atomic force microscopy. We hypothesized that amyloid state of RopB protein could be associated with the virulence (ability to invade and colonize host organism) of *R. leguminosarum* and could contribute to host plant colonization. To analyze the interrelation between RopB amyloidogenesis and *Rhizobium*-legume symbioses establishment we compared amyloid formation by RopB in differentiated bacteroids from effective nodules and undifferentiated bacteroids from ineffective (which are unable to fix nitrogen) nodules. Our data contribute to the understanding of the biological functions of the RopB protein and the involvement of the amyloid state in the symbiotic interactions.

## 2 Materials and methods

### 2.1 *In vitro* experiments

#### 2.1.1 Protein production and purification

For RopB protein production, the pLATE–RopB plasmid (Kosolapova et al., 2019) was used. This vector contains a construct encoding RopB fused with a 6x-His tag under the control of an inducible T7 promoter. For protein production, we used the *E. coli* strain BL21 (DE3) (Studier and Moffatt, 1986). Cells were grown at 37°C in the 2TY medium with ampicillin (1.6% (w/v) tryptone, 1.0% (w/v) yeast extract, 5.0% (w/v) NaCl, 0.01% (w/v) ampicillin (Helikon, Moscow, Russia)). Isopropyl β-D-1-thiogalactopyranoside (IPTG, Thermo Fisher, Waltham, MA, USA) was added to the final concentration of 0.1 mM to induce protein overproduction, and the cells were collected after 6 h of growth with IPTG.

Proteins were purified in denaturing conditions (in the presence of 8 M urea) according to previously published protocols (Serio et al., 1999) with modifications (Kosolapova et al., 2019): a one-step purification procedure with Ni-NTA (nitrilotriacetic acid) agarose (Invitrogen, Carlsbad, CA, USA). Proteins were concentrated and precipitated by adding five volumes of ethanol and stored at −80°C.

#### 2.1.2 The preparation of amyloid fibrils *in vitro*

For the preparation of RopB and vicilin amyloids, the proteins in concentration 0.5 mg/mL were dissolved in 50% 1,1,1,3,3,3-Hexafluoro-2-propanol (HFIP, Sigma-Aldrich, Saint-Louis, MO, USA) and incubated for seven days at 37°C as previously described (Kosolapova et al., 2019; Antonets et al., 2020). Afterwards, the HFIP was evaporated under a stream of nitrogen, and the samples were diluted with Milli-Q water to the original volume and stirred for an additional 7 days at 37°C. These conditions were also used for experiments with seeding. “Seeds” prepared based on the pre-incubated RopB or vicilin aggregates were added to the samples at the beginning of fibrillogenesis at 5% (v/v) concentration.

#### 2.1.3 X-ray diffraction analysis

For x-ray diffraction analysis fibrils (2 mL of 1 mg/mL solution) were lyophilized by using the FreeZone 1L lyophilizer (Labconco, Kansas City, MO, USA). The freeze-dried samples (2 mg) were dissolved in 20 µL of the Milli-Q water (to the final concentration of ~100 mg/mL). Droplets of these preparations were placed between the ends of wax-coated glass capillaries (~1 mm in diameter) separated approximately by 1.5 mm. The fibril diffraction images of RopB were collected by using a Microstar X-ray generator with HELIOX optics equipped with a Platinum135 CCD detector (X8 Proteum system, Bruker AXS, ZEISS, Oberkochen, Germany). Cu Kα radiation, λ=1.54 Å (1 Å=0.1 nm) was used. The samples were positioned at the right angle to the x-ray beam using a four-axis kappa goniometer (Sambrook et al., 1989; Kosolapova et al., 2019).

#### 2.1.4 Atomic force microscopy

The morphological analysis has been carried out through a Bruker Dimension Icon atomic force microscope (Bruker, ZEISS, Oberkochen, Germany), equipped with a Nanoscope V controller operated in PeakForce mode. The measurements have been performed using N-doped Si probes (Bruker Scanasyst-air). A 5-10 µL portion of the studied suspension of the RopB fibrils was deposited on the freshly cleaved mica, left in a wet atmosphere for 5 min for adsorption and air-dried.

#### 2.1.5 Transmission electron microscopy

A transmission electron microscope Libra 120 (Carl Zeiss, Oberkochen, Germany) was applied to visualize the studied aggregates. Samples were put on the copper grids coated with formvar/carbon films (Electron Microscopy Sciences, Hatfield, PA, USA) and stained by a 1% aqueous solution of uranyl acetate. Immunoelectron microscopy study of bacteroids was performed as described earlier in (Levine et al., 1984; Sacher et al., 2020) with modifications for RopB protein (Kosolapova et al., 2019).

#### 2.1.6 Confocal laser scanning microscopy

Confocal laser scanning microscope Olympus FV 3000 (Olympus, Tokyo, Japan) and oil immersion objective with a 60x magnification, the numerical aperture NA 1.42, and laser with excitation line 405 nm were applied to confirm the staining of RopB aggregates with amyloid-specific fluorescent probe ThT.

#### 2.1.7 Thioflavin T binding

ThT UltraPure Grade (AnaSpec, Fremont, CA, USA) without after-purification was used. ThT-fibrils tested solutions were prepared by equilibrium microdialysis using a Harvard Apparatus/Amika device (Harvard Apparatus, Holliston, MA, USA). Equilibrium microdialysis was performed with a concentration of aggregates of about 0.5 mg/ml and an initial concentration of ThT of about 32 μM. Spectroscopic study of the sample and reference solutions prepared by the proposed approach allowed us to determine the spectral and photophysical characteristics ThT bound to tested amyloids (Kuznetsova et al., 2012b).

#### 2.1.8 Spectral measurements

A U-3900H spectrophotometer (Hitachi, Tokyo, Japan) was applied to collect the absorption spectra of the samples. The absorption spectra of all samples and mixtures of samples with ThT were corrected by light scattering according to the standard procedure (Vladimirov and Litvin, 1964).

Fluorescence spectra of the samples were measured using a Cary Eclipse spectrofluorimeter (Varian, Palo Alto, CA, USA). Fluorescence of ThT was excited at a wavelength of 440 nm and recorded at a wavelength of 480 nm. The recorded values of fluorescence intensity were corrected on the primary inner filter effect (Fonin et al., 2014).

Far-UV CD spectra (190 – 260 nm) were measured by a J-810 spectropolarimeter (Jasco, Easton, MD, USA) using a 1 mm path length cell. The secondary structure content of the RopB samples was estimated by the BeStSel web server (Micsonai et al., 2018) as it predicted well the secondary structure of amyloid aggregates enriched by β-sheets (Wang et al., 2021). The server allows estimating the content in α-helices (regular and distorted), β-sheets (parallel β-sheets are distinguished from antiparallel ones), β-turns, and others (which includes 3-10-helix, bend, and unordered structure).

Fluorescence decay curves were recorded by a spectrometer FluoTime 300 (PicoQuant, Berlin, Germany) with the Laser Diode Head LDH-C-440 (λ_ex_ = 440 nm). The fluorescence of ThT was registered at λ_em_ = 490 nm. The measured emission decays were fit to a multiexponential function using the standard convolute-and-compare nonlinear least-squares procedure (O’Connor and Phillips, 1984). In this method, the convolution of the model exponential function with the instrument response function (IRF) was compared to the experimental data until a satisfactory fit was obtained. The fitting routine was based on the nonlinear least-squares method. Minimization was performed according to Marquardt (Marquardt, 1963).

### 2.2 *In vivo* based studies

#### 2.2.1 *Rhizobium leguminosarum* bv. *viciae* strain and cultivation conditions

*Rhizobium leguminosarum* bv. *viciae* RCAM1026 strain (Afonin et al., 2017) from the Russian Collection of Agricultural Microorganisms (RCAM) of the All-Russia Research Institute for Agricultural Microbiology (St. Petersburg, Russia) was used. For analysis of RopB localization in nodules, 3841 strain (Glenn et al., 1980) was used. For inoculation, bacteria were cultivated on solid medium №79 (0.5 g/L K_2_HPO_4_, 0.2 g/L MgSO_4_ x 7H_2_O, 0.1 g/L NaCl, traces of CaCO_3_, 10 g/L mannitol, 0.4 g/L yeast extract, 15 g/L agarose) at 28°C.

Free-living culture of *R. leguminosarum* bv. *viciae* used in experiments was grown in liquid TY medium (5 g/L tryptone, 3 g/L yeast extract, 1.3 g/L CaCl_2_ x 6H_2_O) at 28°C with shaking (170 rpm) overnight.

#### 2.2.2 *Pisum sativum* L. plant material and growing conditions

The pea (*P. sativum* L.) wild-type line Sprint-2 (Fix^+^ phenotype) and corresponding mutant line Sprint-2Fix^-^ (*sym31*) (Fix^-^ phenotype) from the collection of the All-Russia Research Institute for Agricultural Microbiology (St. Petersburg, Russia) were used (Borisov et al., 1997; Malovichko et al., 2020). The Fix^+^ line is characterized by determinate growth, early flowering start, and early seed maturation. The Fix^-^ line is a descendant of the Fix^+^ line that forms ineffective root nodules (unable to fix nitrogen) with undifferentiated bacteroids (Borisov et al., 1992; 1997). Mutant Fix^-^ line has significantly lower growth parameters than those of the wild-type line Fix^+^ and shows signs of nitrogen deficiency (**Supplementary Figure 1A**; **Supplementary Table 1**). The wild-type plants produced large pink nodules, while nodules of the mutant plants were small and white, and their number was more than 2 times that of the wild-type line (**Supplementary Figures 1B, C**; **Supplementary Table 1**).

Pea seeds were surface disinfected for 20 min in 98% sulfuric acid, then thoroughly rinsed with sterile water and soaked for 4 hours in the suspension of *R. leguminosarum* bv. *viciae* RCAM1026 strain (10^6^ CFU/mL). To obtain nodules, Fix^+^ and Fix^-^ plants were grown in sterile quartz sand in a constant environment chamber (model VB 1514, Vötsch, Germany) at 16/8 h and 21/19°C day/night regime, 75% relative humidity, and around 10,000 lux illumination, with the plant nitrogen-free nutrient solution supplement (Borisov et al., 1997).

#### 2.2.3 Bacteroid extraction from pea nodules

For bacteroid extraction root nodules were collected after 25 days of growth after inoculation. Bacteroids for CR staining, analysis of detergent resistance, and immunoelectron microscopy were extracted with the usage of the sucrose-gradient method, as described previously (Catalano et al., 2004; Vedam et al., 2006) with modifications. In brief, nodules were suspended in ice-cold Tris-HCl/sucrose buffer (50 mM Tris-HCl pH 8.0, 0.5 M sucrose supplemented with dithiothreitol and proteinase inhibitor) and crushed with a mortar and pestle. The crushed nodules were filtered through miracloth and Tris-HCl-sucrose buffer was added to the filtrate. The resulting solution was centrifuged for 1 min at 10,000 g. The pellet was resuspended in Tris-HCl buffer and suspension was loaded onto sucrose cushions (ice-cold 50 mM Tris-HCl pH 8.0, 1.5 M sucrose) and centrifuged for 30 s at 5,000 g. The top phase was transferred to a new microtube and centrifuged for 2 min at 10,000 g. The pellet was resuspended in Tris-HCl/sucrose buffer, loaded onto sucrose cushions and centrifuged for 5 min at 10,000 g. The pellet containing symbiosomes was washed three times in 500 μl of 0.5 mM Tris-HCl buffer pH 8.0 supplemented with dithiothreitol and proteinase inhibitor (Sigma Aldrich, St. Louis, MO, USA) and centrifuged. The final pellets were resuspended in 1.5 mL of the 0.5 M Tris-HCl buffer pH 8.0.

#### 2.2.4 Analysis of detergent and protease resistance of protein aggregates and immunodetection

To detect intact RopB protein extracted from *R. leguminosarum* cells, rabbit anti-RopB (PrimeBioMed LLC, Moscow, Russia) antibody and secondary goat anti-rabbit IgG (H+L) antibody (Thermo Scientific, Waltham, MA, USA) were used in the dilutions 1:1,000 and 1:20,000, respectively. Visualization of protein signals by the enhanced chemiluminescence (ECL) PrimeWestern Blotting Detection reagent (GE Healthcare, Chicago, IL, USA), and Bio-Rad ChemiDoc™ hardware and software (Bio-Rad, Hercules, CA, USA) was used for imaging. All samples at the appropriate stage of sample preparation (before adding detergents) underwent concentration equalization near the value of 1 mg/mL of total protein. Measurements were taken using the Qubit 3.0 Fluorometer (Invitrogen, Carlsbad, CA, USA).

#### 2.2.5 Congo red staining and microscopic analysis

For the polarized light microscopy, bacteroids and free-living culture cells were placed onto the slide, dried on air, and stained with the water-based solution of CR (10 mg/mL). The samples were air-dried and washed from the excessive dye with distilled water. Microscopy was performed with the usage of Axio Imager A2 transmitted light microscope (Carl Zeiss, Oberkochen, Germany) equipped with a Plan-Neofluar 20×/0.5 Pol objective (Carl Zeiss, Oberkochen, Germany), cross-polarizers for polarization microscopy and Colibri 7 fluorescent light-emitting diode (LED) light source (Carl Zeiss, Oberkochen, Germany) for fluorescent analysis.

#### 2.2.6 Analysis of RopB localization in root nodules

For analysis of the localization of RopB protein, the nodules of the wild-type and mutant pea lines harvested 14 days after inoculation and were fixed in 2.5% (v/v) glutaraldehyde (Sigma-Aldrich, St. Louis, Missouri, USA) in 0.06 M phosphate buffer, pH 7.2 and subjected to sample preparation as described earlier (Tsyganova et al., 2019). A glancing cut on one side of a nodule was made to achieve better penetration of the fixative. After vacuum infiltration, floating nodules were discarded, and the fixative was replaced with a fresh solution. After overnight incubation at 4°C, nodules were dehydrated in an ascending ethanol series at –35°C and infiltrated and embedded in Lowicryl K4M resin (Electron Microscopy Sciences, Hatfield, PA, USA) by UV polymerization at –20°C.

For transmission electron microscopy, 90-nm-thick ultrathin sections were obtained on a Leica EM UC7 ultramicrotome (Leica Microsystems, Vienna, Austria) and collected on gold grids coated with formvar and carbon (Electron Microscopy Sciences, Hatfield, PA, USA). After blocking with 50 mM glycine in PBS for 15 min and in blocking buffer (5% BSA, 0.1% cold water fish skin (CWFS) gelatin, 5-10% normal goat serum, pH 7.4) for 30 min, the sections were washed by passing through five drops of 0.1% BSA-C in PBS and incubated overnight at 4°C with primary antibody (rabbit anti-RopB (PrimeBioMed LLC, Moscow, Russia)) at dilution 1:100 in 0.1% BSA-C. After washing, the sections were passed through five drops of 0.1% BSA-C, incubated with a secondary antibody, goat anti-rabbit conjugated with 10-nm colloidal gold (Amersham International, Amersham, Buckinghamshire, UK), and diluted 100 in 0.1% BSA-C in PBS for 4 h at room temperature. The grids containing sections were then washed three times in PBS for 20 min, twice in water for 30 min, and counterstained in 2% (w/v) aqueous uranyl acetate for 30 sec. All solutions were filtered before use and filter-sterilized deionized water was used throughout the experiment. The samples were examined and photographed in transmission electron microscopes JEM-1400 (JEOL Corp., Tokyo, Japan) equipped with a Veleta CCD camera (Olympus-SIS, Münster, Germany) and JEM-2100HC (JEOL Corp., Tokyo, Japan) equipped with a Gatan UltraScan 4000 (Gatan, Pleasanton, CA, USA) at 80 kV.

The specificity of the immunogold labelling procedures was tested by several negative controls (**Supplementary Figure 2**). Negative controls were treated either with (i) non-specific secondary antibody (goat anti-mouse IgG) or (ii) gold conjugated secondary antibody (goat anti-rabbit IgG) without the primary antibody.

For statistical analysis, at least 5 different samples of nodules and at least 40-50 sectioned symbiosomes for each variant were examined. Morphometrical data were obtained as described by (Tsyganova et al., 2019). Briefly, the area of symbiosomes and the number of gold particles in it were estimated, then the number of gold particles per unit area was calculated. The areas and the number of gold particles were measured using software Zen 2 Core version 2.5 (Carl Zeiss, Oberkochen, Germany). The data were presented as the number of gold particle/μm^2^. Statistically significant differences were determined by Kruskal–Wallis test and Dunn’s *post hoc* test using R software (R Core Team; http://www.R-project.org/).

#### 2.2.7 PSIA proteomic assay

For identification of potentially amyloidogenic proteins, nodules were ground with a pestle and mortar and suspended in phosphate-buffered saline (PBS, pH 7.4, Helicon, Moscow, Russia) with a 3% sarcosyl (sodium lauroyl sarcosinate) and protease inhibitors cocktail (Sigma Aldrich, St. Louis, MO, USA). The resulted suspension was subjected to PSIA (Proteomic Screening and Identification of Amyloids) approach, which was performed as described previously (Antonets et al., 2016; Nizhnikov et al., 2016). At the initial step, the fraction of protein polymers resistant to treatment with cold 3% sarcosyl was isolated. Then, the polymeric fraction was solubilized by boiling with 1% sodium dodecyl sulfate (SDS) (Amresco, Solon, OH, USA). The solubilized fraction was purified from detergents and salts with HiPPR™ Detergent Removal Spin (Thermo Fisher Scientific, Waltham, MA, USA) and Zeba™ Spin Desalting Columns (Thermo Fisher Scientific, Waltham, MA, USA).

Proteins were cleaned from components of lysis buffer by acetone precipitation (Electron Microscopy Sciences, Hatfield, PA, USA) and resuspended in 8 M Urea/50 mM ammonium bicarbonate (Sigma Aldrich, St. Louis, MO, USA). Then the protein concentration was measured by Qubit 4.0 fluorometer (ThermoFisher Scientific, Waltham, MA, USA) with QuDye Protein Quantification Kit (Lumiprobe, Moscow, Russia) according to the manufacturer recommendations.

The samples (5 μg) were incubated for 1 h at 37°C in 5 mM 1,4-dithiothreitol (Sigma Aldrich, St. Louis, MO, USA) with subsequent incubation in 15 mM iodoacetamide for 30 min in the dark at room temperature (Sigma Aldrich, St. Louis, MO, USA). Next, the samples were diluted with seven volumes of 50 mM ammonium bicarbonate and incubated for 16 h at 37°C with 100 ng of trypsin (ratio 1:50; “Trypsin Gold”, Promega, Madison, WI, USA). Finally, the samples were evaporated in Labconco Centrivap Centrifugal Concentrator (Labconco, Kansas City, MO, USA), dissolved in water (“LC-MS” grade; LiChrosolv, Merck, Darmstadt, Germany) with 0.1% formic acid (“for LC-MS LiChropur”; Merck, Darmstadt, Germany) and desalted with C18 ZipTip (Millipore-Sigma, Burlington, MA, USA) according to the manufacturer recommendations. Desalted peptides were evaporated and dissolved in 20 μL of water/0.1% formic acid for further LC-MS/MS analysis.

Approximate 500 ng of peptides were used for shotgun proteomics analysis by HPLC-MS/MS with ion mobility in TimsToF Pro mass spectrometer (Bruker Daltonics, Bremen, Germany) with nanoElute UHPLC chromatograph (Bruker Daltonics, Bremen, Germany). UHPLC was performed in the two-column separation mode with Acclaim™ PepMap™ 5 mm Trap Cartridge (Thermo Fisher Scientific, Waltham, MA, USA) and Bruker Fifteen separation column (C18 ReproSil AQ, 150 mm × 0.75 mm, 1.9 µm, 120 A; Bruker Daltonics, Bremen, Germany) in gradient mode with 400 nL/min flow rate and 40°C. Phase A was water/0.1% formic acid, phase B was acetonitrile/0.1% formic acid (“LC-MS” grade; LiChrosolv, Merck, Darmstadt, Germany). The gradient was from 2% to 30% phase B for 42 min, then to 95% of phase B for 6 min with a subsequent wash with 95% phase B for 6 min. Before each sample, trap and separation columns were equilibrated with 10 and 4 column volumes respectively.

CaptiveSpray ion source was used for electrospray ionization with 1600 V of capillary voltage, 3 L/min N_2_ flow, and 180°C source temperature. The mass spectrometry acquisition was performed in DDA-PASEF mode with a 0.5 s cycle in positive polarity with the fragmentation of ions with at least two charges in m/z range from 100 to 1700 and ion mobility range from 0.85 to 1.30 1/K0.

Protein identification was performed in PEAKS software (v. 10.6 build 20201221; a license granted to St. Petersburg State University; Bioinformatics Solutions Inc., Waterloo, ON, Canada) using UniProtKB rewieved (Swiss-Prot) database limited to *Pisum sativum* L. (Taxon ID: 3888) and *Rhizobiaceae* family (Taxon ID: 82115) and protein contaminants library for DDA proteomics (Yao et al., 2022). The search parameters were: parent mass error tolerance of 10 ppm and fragment mass error tolerance of 0.05 ppm, protein and peptide FDR less than 0.9%, and three possible missed cleavage sites. Cysteine carbamidomethylation was set as a fixed modification. Methionine oxidation, acetylation of protein N-term, asparagine, and glutamine deamidation were set as variable modifications. Identified proteins were annotated with Gene Ontology (GO) terms (http://geneontology.org/) in accordance with the UniProt (https://www.uniprot.org/) database. GO terms were retrieved with UniprotR package (Soudy et al., 2020). The mass spectrometry proteomics data have been deposited to the ProteomeXchange Consortium *via* the PRIDE (Perez-Riverol et al., 2022) partner repository with the dataset identifier PXD035724 and 10.6019/PXD035724.

## 3 Results

### 3.1 RopB forms *bona fide* amyloids *in vitro*

Previously, we have demonstrated such amyloid properties of RopB protein aggregates as fibrillar morphology, affinity to amyloid-specific dyes and resistance to treatment with ionic detergents and proteases (Kosolapova et al., 2019). Here, we performed several experiments to finally confirm an ability of RopB to adopt *bona fide* amyloid state.

**Figure 1:**
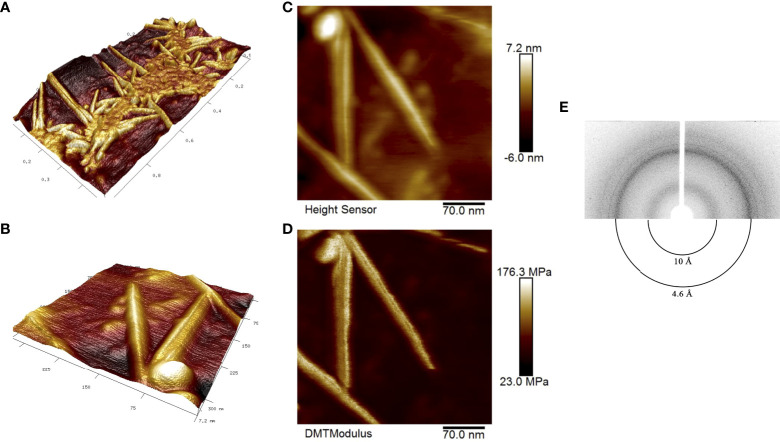
Structural properties of the RopB amyloid fibrils obtained *in vitro*. **(A)** and **(B)** high-resolution 3D landscape reconstruction of the RopB fibrils samples by AFM analysis at different magnifications: general view in microns **(A)** and individual fibrils, nm **(B)**. **(C)** Height Sensor image shows the RopB fibrils height. Scale bar are shown, nm. **(D)** DMT modulus image shows rigidity of the fibrils. Scale bar are shown, MPa. **(E)** X-ray diffraction patterns of the lyophilized RopB fibrils formed *in vitro*. Shown are reflections in angstroms (Å).

We obtained RopB fibrils as described in ‘Materials and Methods’ and subjected samples to atomic force microscopy (AFM). **Figures 1A**, **B** show a general view of the fibril sample and individual fibrils, respectively, in 3D landscape reconstruction at different magnifications. Based on these data we may state that AFM analysis confirms linear and unbranched morphology of the RopB fibrils typical for the amyloid fibrils. Due to the limited capability of the AFM probe to measure the fibril width, this parameter can be determined using the AFM ‘Height Sensor’ (**Figure 1C**), where the fibril width is defined as 7.2 ± 0.7 nm. According to previously obtained transmission electron microscopy (TEM) data, the average fibril width was 8.3 ± 1.2 nm (Kosolapova et al., 2019), the difference is not significant. A slight discrepancy with the TEM data can be explained by the fact that, when stained with a dye (uranyl acetate) for TEM analysis, the fibrils seem to flow along the sides, which visibly makes them slightly wider. Therefore, we believe that the TEM and AFM data are consistent with each other. The fibrils also look very indicative and clear in the rigidity DMT (Derjaguin-Muller-Toporov) modulus (**Figure 1D**), in which only rigid objects are detected and the “flexible” monomeric protein leaves (Derjaguin et al., 1975).

To finally confirm amyloid properties of the RopB fibrils, we analyzed their x-ray diffraction patterns. The RopB samples had undergone desalting by dialysis prior experiments. The x-ray diffraction experiments demonstrated circular x-ray reflections 4.6 Å and 10 Å for the RopB fibrils (**Figure 1E**, **Supplementary Figure 3**). These reflections are indicative of the *bona fide* amyloid fibrils arising from their cross-β spatial structure and are supposed to indicate: (i) periodicity of the hydrogen-bonded β-strands (4.6 Å) and (ii) stacking of the β-sheets near parallel to the fiber axis (10 Å) (Sunde and Blake, 1997).

Thus, we may conclude that RopB protein of *R. leguminosarum* forms fibrils that satisfy all the criteria of *bona fide* amyloids.

### 3.2 RopB forms amyloid fibrils *in vivo* during colonization of pea nodules by *R. leguminosarum*

Previously, we have shown that free-living culture of *R. leguminosarum* forms extracellular RopB fibrils in the stationary growth phase (Kosolapova et al., 2019). Here, we decided to study whether the formation of RopB fibrils occurs during *R. leguminosarum* interaction with pea (*P. sativum* L.).

**Figure 2:**
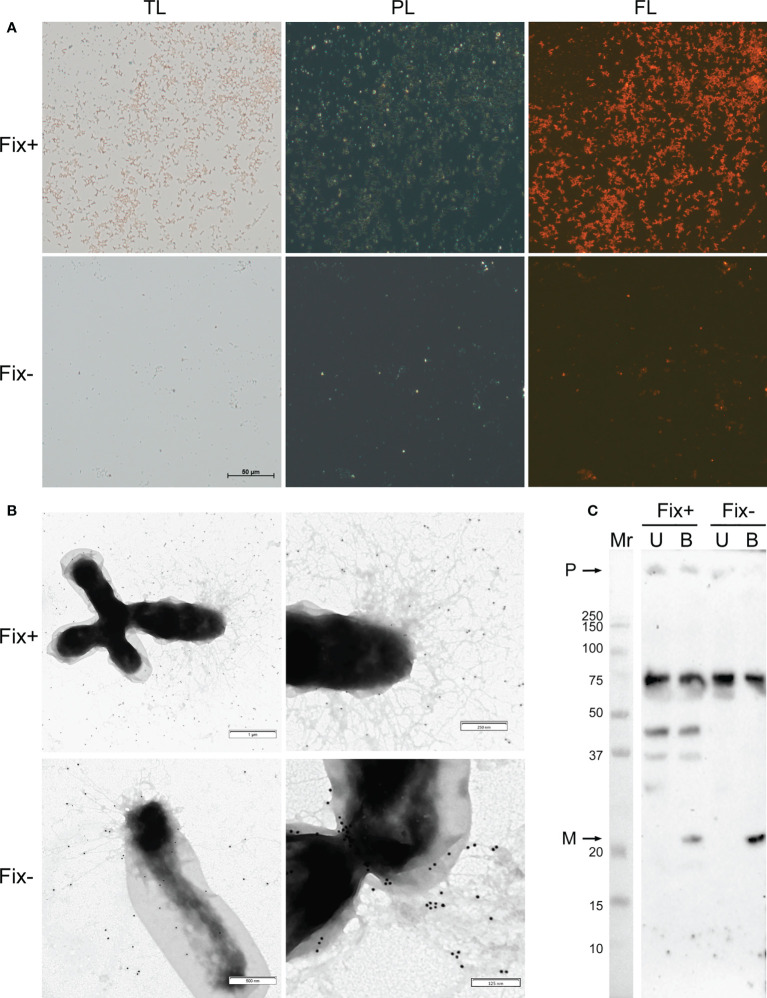
Amyloid properties of RopB protein in *R. leguminosarum* bacteroids extracted from nodules of *P. sativum* L. formed by wild-type Sprint-2 (Fix^+^ phenotype) and mutant Sprint-2Fix^-^ (*sym31*) pea lines. **(A)** Transmitted light (TL), polarization (PL) and fluorescent (FL) microscopy of Congo red stained bacteroids. All images have the same magnifications; scale bar is shown in µm. **(B)** Immunoelectron images of the bacteroids labelled with anti-RopB antibody visualized by gold-conjugated secondary antibody. Scale bars are indicated. For negative control see **Supplementary Figure 4**. **(C)** Detection of the RopB aggregate formation by sodium dodecyl sulfate-polyacrylamide gel electrophoresis (SDS–PAGE) followed by western blot hybridization. Samples treated with cold (U, unboiled) and hot (B, boiled) 2% SDS are shown. “M” – monomers, “P” – polymers, “Mr” – molecular weight marker. Molecular weights (kDa) are indicated.

For analysis of amyloid properties of RopB protein *in vivo* during the symbiotic interactions we extracted bacteroids formed by *R. leguminosarum* bv. *viciae* RCAM1026 from nodules of pea wild-type line Sprint-2 (Fix^+^ phenotype) and ineffective mutant line Sprint-2Fix^-^ (*sym31*) (Fix^-^ phenotype) (Borisov et al., 1992; 1997). Extracted *R. leguminosarum* bacteroids were stained with the amyloid-specific CR dye. Bacteroids from both lines bound CR and exhibited fluorescence in the fluorescent light and birefringence in the polarized light indicating presence of amyloid structures (**Figure 2A**). Nevertheless, differentiated bacteroids extracted from the wild-type nodules were more congophylic than relatively undifferentiated bacteroids from mutant nodules (**Figure 2A**). This effect can be explained by the fact that the size of Fix^+^ bacteroids is significantly bigger than those of Fix^-^. Also, almost all Fix^+^ bacteroids were CR-positive while only several Fix^-^ bacteroids were congophylic (**Figure 2A**). Using transmission immunoelectron microscopy we observed fibrillar structures on the surface of *R. leguminosarum* bacteroids from both wild-type and mutant pea lines; those fibrils bound anti-RopB antibodies (**Figure 2B**; **Supplementary Figure 4**). Thus, *R. leguminosarum* bacteroids produce RopB-containing fibrils at their surface and demonstrate birefringence indicating the presence of amyloids.

Another important property of amyloids is the resistance to treatment with ionic detergents. To analyze the formation of aggregates by RopB we extracted total protein fractions from *R. leguminosarum* bacteroids isolated from the wild-type and mutant nodules. Samples were either incubated with 2% SDS at room temperature or boiled with 2% SDS. The results of western blot hybridization demonstrate that RopB forms aggregates resistant to treatment with 2% SDS in both variants of bacteroids analyzed (**Figure 2C**). Nevertheless, RopB aggregates from bacteroids isolated from the wild-type line are resistant to boiling with SDS, while aggregates from non-differentiated bacteroids from the *sym31* mutant line completely solubilized after boiling with SDS (**Figure 2C**). Notably, bacteroids from the wild-type line contained specific oligomeric form of RopB (**Figure 2C**) that may either reflect its membrane β-barrel state or correspond to a specific structural subunit of the RopB fibrils partially explaining the difference in the resistance to boiling with SDS between RopB fibrils from Fix^+^ and Fix^-^ bacteroids.

**Figure 3:**
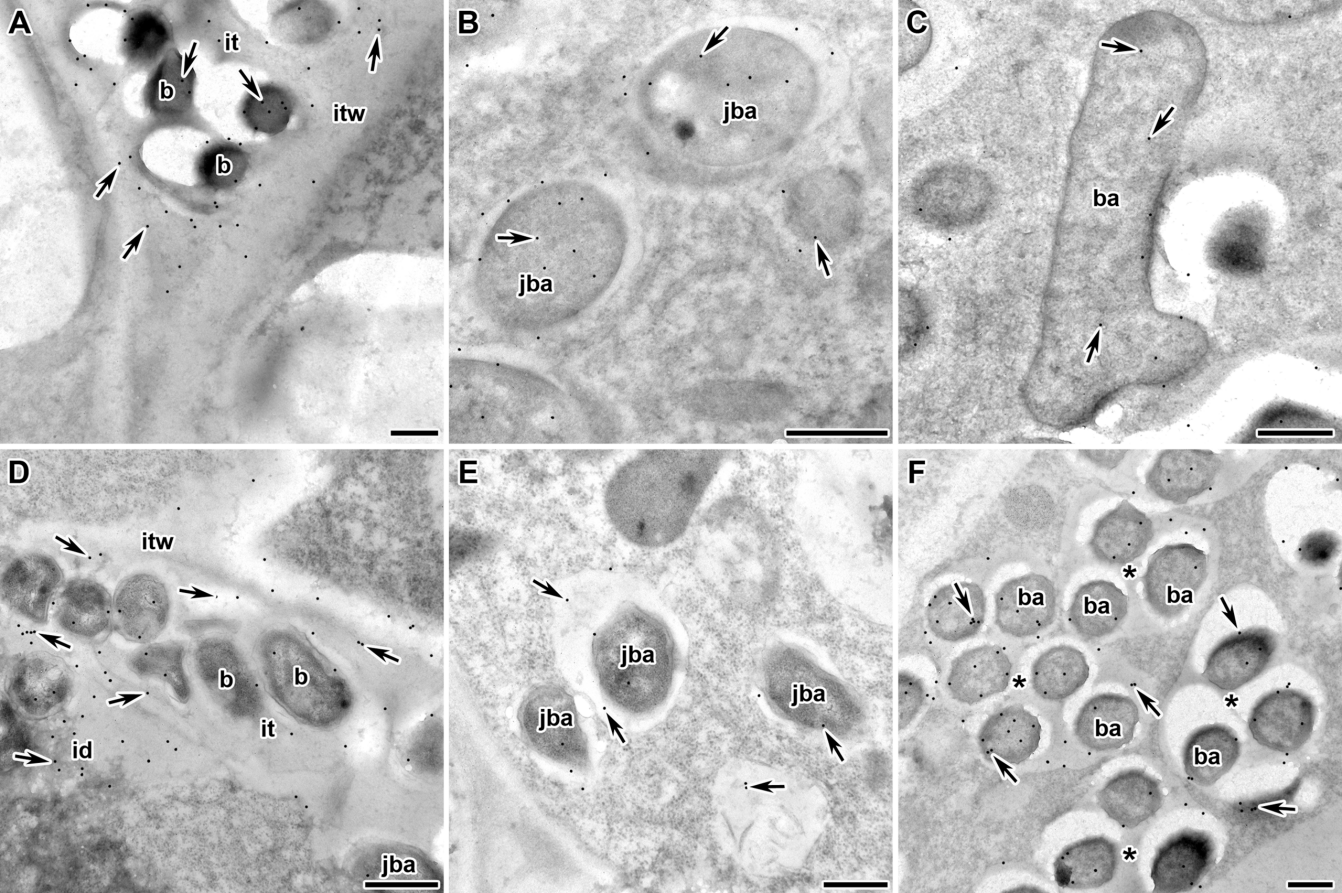
Immunogold localization of RopB protein in the nodules of *P. sativum* L. in wild-type line Sprint-2Fix^+^ **(A–C)** and mutant line Sprint-2Fix^-^(*sym31*) **(D–F)**. The RopB label is in the infection thread **(A, D)**, juvenile symbiosomes **(B, E)**, mature symbiosomes **(C)** and multibacteroid symbiosomes characteristics for mutant line Sprint-2Fix^-^(*sym31*) **(F)**. it – infection thread, itw – infection thread wall, id – infection droplet, b – bacteria, ba – bacteroid, jba – juvenile bacteroid, * – multibacteroid symbiosome, arrows indicate gold particles. Scale bars are equal to 500 nm. For negative control see **Supplementary Figure 2**.

Next, we analyzed the localization of RopB protein in wild-type and mutant pea nodules formed by *R. leguminosarum* bv. *viciae* strain 3841 with immunoelectron microscopy. Immunogold localization of RopB showed that this protein was distributed evenly in bacteria and matrix inside infection threads in nodules of both studied genotypes suggesting the presence of extracellularly produced RopB state (**Figures 3A**, **D**; **Supplementary Figure 2**). In addition, the label was found in the infection thread walls, but in much smaller quantities. In the infected cells, the RopB protein label was localized in symbiosomes, i.e., in bacteroids, peribacteroid space and on the symbiosome membrane (**Figures 3B, C**, **F**).

The amount of RopB protein label seems to be higher in the juvenile symbiosomes of the wild-type line (15.9 ± 2.0) (**Figure 3B**) but significantly decreased in the mature symbiosomes (9.1 ± 1.5, p < 0.05) (**Figure 3C**). In the *sym31* mutant line in juvenile bacteroids, in contrast to the wild type, the amount of the RopB protein label was low (6.9 ± 1.6) (**Figure 3E**), but in multibacteroid symbiosomes, the amount of the label significantly increased (12.9 ± 1.7, p < 0.05) (**Figure 3F**). The number of gold particles in multibacteroid symbiosomes in the mutant *sym31* is almost the same as that in juvenile symbiosomes in nodules of the wild-type pea.

Taken together, we may conclude that *R. leguminosarum* bacteroids produce extracellular RopB amyloids *in vivo* in pea nodules.

### 3.3 A landscape of potentially amyloidogenic proteins in pea root nodules

The next question was which proteins of pea and rhizobia, in addition to RopB, could adopt amyloid state in pea nodules? To reveal potentially amyloidogenic proteins in pea nodules we used previously developed PSIA proteomic approach. This method is based on the identification of proteins that form aggregates and complexes resistant to treatment with ionic detergents, which is one of the key properties of amyloids (Nizhnikov et al., 2016). To characterize the landscape of detergent-resistant proteins in nodules, we performed the PSIA analysis of potentially amyloidogenic proteins in the proteome of nodules formed by the wild type and the *sym31* mutant, inoculated with *R. leguminosarum* bv. *viciae* RCAM1026.

**Figure 4:**
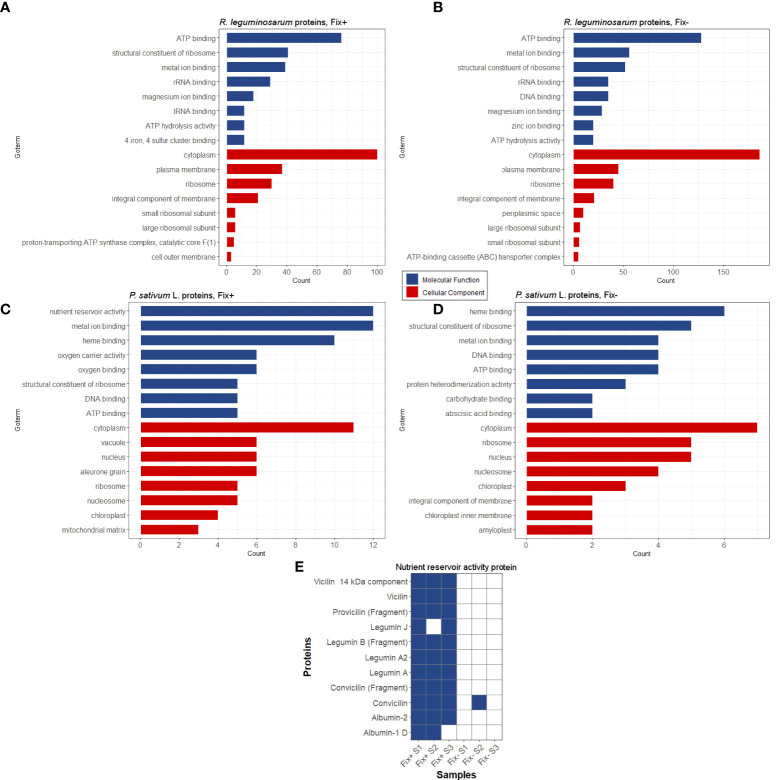
Potentially amyloidogenic proteins of Fix^+^ and Fix^-^ root nodules formed by *R. leguminosarum* **(A)** and **(B)**, *P. sativum* L. **(C)** and **(D)**, respectively. Top-8 GO annotations of “Molecular Processes” and “Cellular Component” categories of identified proteins based on UniProt data are shown. **(E)** Potentially amyloidogenic proteins of *P. sativum* L. with “nutrient reservoir” activity identified in protein samples obtained from wild-type and *sym31* mutant nodules. Proteins identified in particular samples (S1-S3) and Fix status of nodules are shown.

We have extracted protein aggregates and complexes resistant to treatment with 3% ionic detergent sodium N-lauroyl sarcosinate (sarcosyl) from pea nodules and subjected them to trypsinolysis followed by high-performance liquid chromatography coupled with mass-spectrometry (for a detailed description, see “Materials and Methods”). As a result, we identified 300 proteins of *Rhizobiaceae* and 63 proteins of *P. sativum* L. in the sarcosyl-insoluble fraction of Fix^+^ pea nodules and 512 proteins of *Rhizobiaceae* and 42 proteins of *P. sativum* L. in the sarcosyl-insoluble fraction of Fix^-^ pea root nodules. Sarcosyl is a milder detergent in comparison with SDS (Nizhnikov et al., 2014), which partially explains a relatively high number of identified sarcosyl-insoluble proteins. The identified proteins were annotated with GO terms (**Figures 4A–D**). The majority of identified proteins of *R. leguminosarum* from both wild-type (Fix^+^ phenotype) and mutant (Fix^-^ phenotype) nodules are localized in the cytoplasm and possess ATP-binding activity (**Figures 4A, B**; **Supplementary Table 2**). Other highly abundant groups of identified proteins of *R. leguminosarum* are present by ribosomal proteins and proteins with different binding activities. Within identified proteins only three are localized in the outer membrane: RopB (**Supplementary Figure 5**), RopA (whose amyloidogenic properties have been demonstrated in (Kosolapova et al., 2019)), and peptidoglycan-associated lipoprotein (Pal), a component of the Tol-Pal system involved in the control of the late stages of cell division (Krol et al., 2020). The periplasmic bacterial proteins including another component of Tol-Pal system, Pal-associated TolB protein (which was identified in only one sample) (Clavel et al., 1998), were specific for mutant nodules. Such a formation of detergent resistant aggregates by different periplasmic proteins of undifferentiated bacteroids from Fix^-^ nodules might suggest for probable proteostasis defects occurring in the ineffective nodules (**Supplementary Figure 6**) as disturbed proteostasis can emerge in form of protein aggregation and misfolding (Laskowska et al., 2019). Nitrogenase components NifE and NifH were solely identified in Fix^+^ samples agreeing with their nitrogen fixing activity. The largest functional group of identified *P. sativum* L. proteins from Fix^+^ nodules was the “nutrient reservoir activity” proteins localized in vacuole (**Figures 4C, D**). These group includes major storage globulins (vicilin (**Supplementary Figure 7**), legumin, convicilin) and albumins (Dziuba et al., 2014). Notably, “nutrient reservoir activity” proteins were identified only in the detergent-resistant fraction from wild-type nodules but not in mutant nodules suggesting their involvement in the plant-microbial interactions (**Figures 4C–E**). Interestingly, one of pea “nutrient reservoir activity” proteins identified in the detergent resistant protein fraction from wild-type nodules is vicilin (**Figure 4E**), whose amyloid properties have been demonstrated previously (Antonets et al., 2020). Other abundant groups of identified *P. sativum*L. proteins contained ribosome components and proteins with different binding activities (**Figures 4C, D**).

Overall, these data suggest that pea nodules contain detergent-resistant aggregates of several known amyloids (vicilin, RopB, RopA) and of a set of proteins that can be considered as potentially amyloidogenic. Interestingly, some identified potentially amyloidogenic proteins were specific for wild-type or mutant nodules indicating significant differences between these two pea lines.

### 3.4 Vicilin cross-seeds fibrillization of RopB *in vitro*

The results of our proteomic analysis in the wild-type pea nodules revealed the presence of detergent-resistant aggregates of pea vicilin and *R. leguminosarum* RopB proteins. Considering that amyloid fibrils of some proteins can specifically seed the amyloidogenesis of others (Chaudhuri et al., 2019; Ren et al., 2019), we decided to test the possibility of cross-induction of the amyloid formation by these proteins.

**Figure 5:**
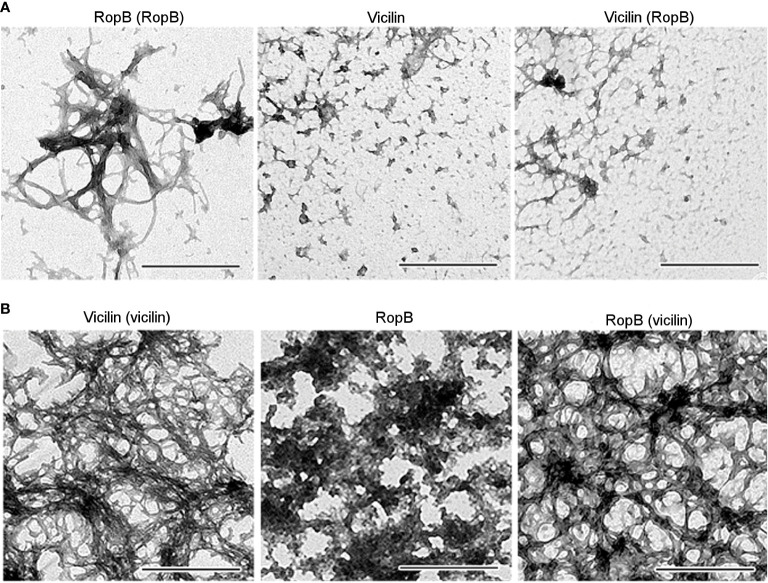
**(A)** TEM images of the RopB fibrils prepared with seeding by pre-incubated RopB aggregates (left panel), vicilin aggregates (middle panel), and vicilin aggregates prepared with cross-seeding by pre-incubated RopB fibrils (right panel). The scale bars are equal to 500 nm. **(B)** TEM images of the vicilin fibrils prepared with seeding by pre-incubated vicilin aggregates (left panel), RopB unstructured aggregates (middle panel), and RopB fibrils prepared with cross-seeding by pre-incubated vicilin amyloids (right panel). The scale bars are equal to 500 nm.

Previously, we selected the optimal conditions for obtaining *bona fide* vicilin (Antonets et al., 2020) and RopB (Kosolapova et al., 2019) amyloid fibrils *in vitro* and characterized their physicochemical properties. Vicilin and RopB amyloids prepared by the developed method (**Figures 5A, B**, left panels) were used to cross-seed each other’s fibrillogenesis. This experiment was carried out under conditions, in which spontaneous fibrillogenesis of vicilin and RopB does not occur, but the proteins can form mainly morphologically unstructured aggregates of different sizes (**Figures 5A, B**, middle panels). The properties of these aggregates were characterized earlier (Kosolapova et al., 2019; Antonets et al., 2020). Transmission electron microscopy data indicate that the presence of a 5% seed of pre-prepared RopB fibrils did not affect the morphology of vicilin aggregates (**Figure 5A**, right panel). At the same time, the vicilin seed leads to a significant change in the morphology of RopB aggregates, which become fibrous unbranched structures prone to agglomeration (**Figure 5B**, right panel), similar in morphology to the seed formed from vicilin (**Figure 5A**, left panel). Next, we analyzed the structure and properties of the obtained clustered fibrils.

**Figure 6:**
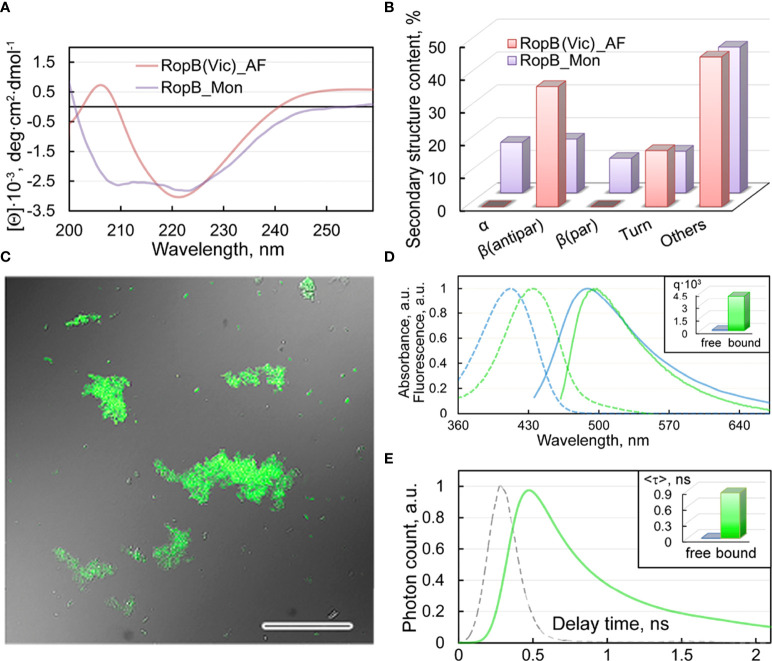
Structure and properties of the obtained RopB fibrils **(A)** CD spectra in the far UV region of RopB in monomeric (purple curve) and fibrillary (pink curve) states. **(B)** Deconvolution of CD spectra of the RopB in monomeric (purple column) and fibrillary (pink column) state using the BeStSel method (Micsonai et al., 2018). The change in the content of α-helices, parallel and antiparallel β-sheets, β-turns, and other structures (includes 3-10-helices, bends, and unordered structures) are shown. **(C)** Confocal microscopy of the RopB fibrils stained with ThT. The overlay of the fluorescence image of the ThT-stained aggregates and the transmitted light image is presented. The scale bar is equal to 50 μm. **(D)** Normalized to unity at the maximum absorption (dotted curves) and fluorescence (straight curves) spectra of ThT in free (blue curves) and bound to RopB aggregates (green curves) states. The Inset shows the values of the fluorescence quantum yield of the dye in free (blue column) and bound to RopB aggregates (green column) states. **(E)** Fluorescence decay curve of the bound to fibrils ThT (green curve) and excitation laser impulse profile (grey curve). The Inset shows the values of the fluorescence lifetime of the dye (in nanoseconds - ns) in free (blue column) and bound to RopB aggregates (green column) states.

The results of studying the obtained RopB aggregates by CD spectroscopy in the far UV region indicate the presence in the CD spectrum of cross-seeded aggregates of a pronounced minimum in the spectral region of 220 nm, which is characteristic of amyloids (**Figure 6A**). Quantitative analysis of the content of secondary structure elements in the sample by the BeStSel web server (Micsonai et al., 2018), indicates a high content (about 37%) of antiparallel β-sheets (**Figure 6B**), which are known to be involved in the formation of the backbone of amyloid fibrils. Our results indicate that the formation of these antiparallel β-sheets is largely due to structural transformations of the parallel β-sheets and α-helices present in the monomeric protein (**Figure 6B**).

Staining of the studied RopB aggregates with the amyloid-specific probe ThT was shown by the confocal laser scanning microscopy (**Figure 6C**). A spectroscopic study (**Figure 6D, E**) of dye-fibrils samples prepared by the equilibrium microdialysis (Kuznetsova et al., 2012a) showed a long-wavelength shift in the absorption spectrum of the fibril-bound dye by more than 20 nm (**Figure 6D**), as well as an increase in the fluorescence quantum yield by several tens of times (**Figure 6D**, Inset) and the lifetime of the excited state (**Figure 6E**, Inset) by 3 orders compared to characteristics of free ThT in an aqueous solution. We observed similar results when studying the interaction of ThT with vicilin amyloids (Antonets et al., 2020), which were used as a seed.

Thus, the obtained results indicate the amyloid-like properties of RopB aggregates prepared in the presence of vicilin amyloids: fibrillar structure, high content of antiparallel β-sheets, and the specificity of their interaction with the ThT fluorescent probe. This means that plant amyloids can modulate amyloidogenesis of rhizobial proteins.

## 4 Discussion

To date, there have been identified about 30 bacterial amyloidogenic proteins (Kosolapova et al., 2020). Amyloids of bacteria perform various physiological functions including biofilm matrix formation, adhesion, and toxins activity regulation (Romero and Kolter, 2014; Cámara-Almirón et al., 2018; Sønderby et al., 2022). Many of those functions are associated with the interactions of bacteria with other organisms (van Gerven et al., 2018). These interactions can be classified into three types: (i) interactions within microbial communities, (ii) pathogenic interactions with multicellular hosts and (iii) symbiotic interactions with multicellular hosts (Kosolapova et al., 2020). However, the data on the role of bacterial amyloids in symbiotic interactions are scarce.

Previously, we analyzed the amyloid properties of outer membrane proteins RopA and RopB from root nodule bacterium *R. leguminosarum in vitro* and demonstrated *in vivo* amyloid formation by these proteins during the stationary growth phase of free-living culture (Kosolapova et al., 2019). In this work, we demonstrated that amyloid fibrils of RopB exhibit typical for amyloids x-ray diffraction pattern (Eanes and Glenner, 1968; Morris and Serpell, 2012) with reflections at 4.6 Å and 10 Å (**Figure 1C**) indicating cross-β spatial structure of the analyzed fibrils. Combining these and previously obtained data (Kosolapova et al., 2019) and results of AFM analysis (**Figures 1A, B**) we can conclude that RopB can adopt *bona fide* amyloid state.

Notably, RopB monomeric protein is predicted to have the β-barrel fold probably acting as the transmembrane protein involved in the outer membrane stabilization (Foreman et al., 2010). Combined with *in vivo* observations such as the presence of RopB-containing fibrils on the surface of bacteroids (**Figures 2**, **3**) we may propose the ability of RopB protein to adopt two different spatial conformations: amyloid fibril and β-barrel. The formation of different spatial forms allows to hypothesize ability of RopB protein to perform diverse functions specifically associated with either its transmembrane β-barrel fold or amyloid state. This hypothesis is supported by the fact that RopB is a part of fibrillar aggregates formed on the surface of both differentiated and undifferentiated bacteroids from the wild-type and mutant nodules, respectively (**Figure 2**). The presence of fibrous matrix surrounding bacteroids in symbiosomes has been observed earlier (Cermola et al., 2000). The fibrillar material has been proposed to be of polysaccharide nature (Cermola et al., 1994). Our findings support the idea that bacteroids are embedded in fibrous matrix composed not only of polysaccharides, but also amyloid proteins.

The analysis of the localization of RopB protein in nodules revealed its presence in bacteroids, peribacteroid space and on the symbiosome membrane of the infected cells (**Figure 3**). Notably, amounts of RopB protein were different in wild-type and mutant nodules. RopB amount increases with the transition from a free-living state to a symbiotic one in juvenile bacteroids in wild-type nodules (**Figure 3**). In this way RopB fibrils could play a certain defense function similarly to other surface biopolymers (Yang et al., 2022), protecting and stabilizing bacteroids at the initial stages of the development. However, with further differentiation, the amount of this protein significantly decreases in mature symbiosomes (**Figure 3**) though even mature bacteroids stained with CR exhibit birefringence in the polarized light and produce fibrils that bind anti-RopB antibody (**Figure 2**) suggesting the presence of RopB amyloids at their surface. An increase in the amount of RopB protein in multibacteroid symbiosomes (containing undifferentiated bacteroids) of the *sym31* mutant probably corresponds to high amount of RopB in juvenile symbiosomes of wild-type (**Figure 3**), indicating a delay in RopB accumulation in the mutant line. Thus, an increase in the RopB production is associated with free-living cell – to bacteroid transition but RopB amyloids form at different stages of bacteroid development suggesting their functional role in this process. These observations are in line with other studies demonstrating the amyloidogenic properties of different β-barrel proteins (Sulatskaya et al., 2021).

Another β-barrel protein whose detergent-resistant aggregates are detected in the root nodules is vicilin, a seed storage protein of *P. sativum* L., that contains two ancient cupin β-barrel domains (Bäumlein et al., 1995; Shutov et al., 1995). We have previously demonstrated the ability of vicilin to form amyloids *in vitro* and *in vivo*, in pea seeds (Antonets et al., 2020). Notably, vicilin and other proteins with nutrient reservoir activity were identified in samples extracted from functional, nitrogen-fixing nodules but not in ineffective nodules with undifferentiated bacteroids (Borisov et al., 1997) (**Figure 4**). The presence of detergent-resistant proteins with nutrient reservoir activity only in effective nodules may highlight the link between nitrogen fixation and aggregation of storage proteins in nodules. Interestingly, vicilin amyloid can cross-seed amyloid formation by RopB protein *in vitro* but not vice versa (**Figure 5**, **6**). Cross-seeding of amyloids is a selective process (Ren et al., 2019; Subedi et al., 2022) suggesting for a possibility of interaction between RopB and vicilin *in vivo.* Functional importance of such probable interaction is unclear, nevertheless, vicilin is known to have a lectin (carbohydrate-binding) activity (Gomes et al., 1997; Rose et al., 2003). Lectins are well known to participate in the specificity of recognition of the nodule bacteria by their plant hosts (Law and Strijdom, 1984; Hirsch, 1999; Danhorn and Fuqua, 2007). Nevertheless, vicilin aggregates were detected only in effective but not in ineffective nodules suggesting that their function is unrelated to bacteria recognition. What is more likely, the interaction (including cross-seeding) between plant and *Rhizobium* amyloids could be involved in the later stages of plant-microbial interactions supporting the bacteroid differentiation and nitrogen fixation. Analysis of RopB protein in other pea ineffective mutants will more accurately identify the stage of nodule development at which the amyloid form of RopB protein is required.

Taken together, in this work we confirmed *bona fide* amyloid properties of RopB amyloids. We showed that RopB amyloids are formed *in vivo* during colonization of plant tissues with rhizobia. This process depends on the particular stage of infection, and RopB amyloids can be cross-seeded by plant amyloid protein vicilin which is also produced in the nodules.

## Data availability statement

The original contributions presented in the study are publicly available. This data can be found here: ProteomeXchange, PXD035724.

## Author contributions

AN, AK, AS, MB, and AT conceived the study and designed the manuscript. AK, MB, AS, MS, OS, AT, and AB performed the experiments. AN, VT, VZ, IT, and KV analyzed the data. AN, AK, AS and MB prepared the manuscript. AK, MB, AS, MS, OS, AT, AB, VT, VZ, IT, KV, and AN critically reviewed the manuscript. All authors contributed to the article and approved the submitted version.

## Funding

The article was made with the support of the Ministry of Science and Higher Education of the Russian Federation in accordance with agreement № 075-15-2021-1055 date September 28, 2021 on providing a grant in the form of subsidies from the Federal budget of Russian Federation. The grant was provided for the implementation of the project: “Mobilization of the genetic resources of microorganisms on the basis of the Russian Collection of Agricultural Microorganisms (RCAM) at the All-Russia Research Institute for Agricultural Microbiology (ARRIAM) according to the network principle of organization”.

## Acknowledgments

The authors thank Bozhana Zainullina and Egor Repkin for help with proteomic experiments and Ekaterina Vasileva for help with cultivation of bacteria. The authors acknowledge the Core Centrum “Genomic Technologies, Proteomics and Cell Biology” (All-Russia Research Institute of Agricultural Microbiology) and Center for Molecular and Cell Technologies (Research Park, St. Petersburg State University) for the equipment provided for use in this work. We are grateful to Zaure Vasileva and Denis Fokin from Carl Zeiss LLC (Moscow, Russia) for their help in performing AFM imaging. We also thank Oksana Belousova for English editing.

## Conflict of interest

The authors declare that the research was conducted in the absence of any commercial or financial relationships that could be construed as a potential conflict of interest.

## Publisher’s note

All claims expressed in this article are solely those of the authors and do not necessarily represent those of their affiliated organizations, or those of the publisher, the editors and the reviewers. Any product that may be evaluated in this article, or claim that may be made by its manufacturer, is not guaranteed or endorsed by the publisher.

## References

[B1] Afonin A. Sulima A. Zhernakov A. Zhukov V. (2017). Draft genome of the strain RCAM1026 *Rhizobium leguminosarum* bv. *viciae* . Genom. Data 11, 85–86. doi: 10.1016/j.gdata.2016.12.003 28053873 PMC5198633

[B2] Antonets K. S. Belousov M. V. Sulatskaya A. I. Belousova M. E. Kosolapova A. O. Sulatsky M. I.. (2020). Accumulation of storage proteins in plant seeds is mediated by amyloid formation. PloS Biol. 18, e3000564. doi: 10.1371/journal.pbio.3000564 32701952 PMC7377382

[B3] Antonets K. S. Volkov K. V. Maltseva A. L. Arshakian L. M. Galkin A. P. Nizhnikov A. A. (2016). Proteomic analysis of *Escherichia coli* protein fractions resistant to solubilization by ionic detergents. Biochem. (Mosc.) 81, 34–46. doi: 10.1134/S0006297916010041 26885581

[B4] Bäumlein H. Braun H. Kakhovskaya I. A. Shutov A. D. (1995). Seed storage proteins of spermatophytes share a common ancestor with desiccation proteins of fungi. J. Mol. Evol. 41, 1070–1075. doi: 10.1007/BF00173188 8587105

[B5] Borisov A. Y. Morzina E. V. Kulikova O. A. Tchetkova S. A. Lebsky V. K. Tikhonovich I. A. (1992). New symbiotic mutants of pea (*Pisum sativum* L.) affecting either nodule initiation or symbiosome development. Symbiosis 14, 297–313.

[B6] Borisov A. Y. Rozov S. Tsyganov V. Kulikova O. Kolycheva A. Yakobi L.. (1994). Identification of symbiotic genes in pea (*Pisum sativum* L.) by means of experimental mutagenesis. Genetika (Russian Fed) 30, 1484–1494.

[B7] Borisov A. Y. Rozov S. M. Tsyganov V. E. Morzhina E. V. Lebsky V. K. Tikhonovich I. A. (1997). Sequential functioning of *Sym-13* and *Sym-31*, two genes affecting symbiosome development in root nodules of pea (*Pisum sativum* L.). Mol. Gen. Genet. 254, 592–598. doi: 10.1007/s004380050456 9197420

[B8] Cámara-Almirón J. Caro-Astorga J. de Vicente A. Romero D. (2018). Beyond the expected: the structural and functional diversity of bacterial amyloids. Crit. Rev. Microbiol. 44, 653–666. doi: 10.1080/1040841X.2018.1491527 30354913

[B9] Catalano C. M. Lane W. S. Sherrier D. J. (2004). Biochemical characterization of symbiosome membrane proteins from *Medicago truncatula* root nodules. Electrophoresis 25, 519–531. doi: 10.1002/elps.200305711 14760646

[B10] Cermola M. Fedorova E. Taté R. Riccio A. Favre R. Patriarca E. J. (2000). Nodule invasion and symbiosome differentiation during *Rhizobium etli*-*Phaseolus vulgaris* symbiosis. Mol. Plant Microbe Interact. 13, 733–741. doi: 10.1094/MPMI.2000.13.7.733 10875334

[B11] Cermola M. Hermann R. Müller M. Taté R. Favre R. (1994). Ultrastructural analysis of *Rhizobium leguminosarum phaseoli* in high-pressure cryofixed ban root nodules. J. Struct. Biol. 113, 142–147. doi: 10.1006/jsbi.1994.1045

[B12] Chaudhuri P. Prajapati K. P. Anand B. G. Dubey K. Kar K. (2019). Amyloid cross-seeding raises new dimensions to understanding of amyloidogenesis mechanism. Ageing Res. Rev. 56, 100937. doi: 10.1016/j.arr.2019.100937 31430565

[B13] Clavel T. Germon P. Vianney A. Portalier R. Lazzaroni J. C. (1998). TolB protein of *Escherichia coli* K-12 interacts with the outer membrane peptidoglycan-associated proteins pal, lpp and OmpA. Mol. Microbiol. 29, 359–367. doi: 10.1046/j.1365-2958.1998.00945.x 9701827

[B14] Dahiya P. Sherrier D. J. Kardailsky I. V. Borisov A. Y. Brewin N. J. (1998). Symbiotic gene *Sym31* controls the presence of a lectinlike glycoprotein in the symbiosome compartment of nitrogen-fixing pea nodules. Mol. Plant Microbe Interact. 11(9), 915–923. doi: 10.1094/MPMI.1998.11.9.915

[B15] Danhorn T. Fuqua C. (2007). Biofilm formation by plant-associated bacteria. Annu. Rev. Microbiol. 61, 401–422. doi: 10.1146/annurev.micro.61.080706.093316 17506679

[B16] Danoff E. J. Fleming K. G. (2015). Aqueous, unfolded OmpA forms amyloid-like fibrils upon self-association. PloS One 10, e0132301. doi: 10.1371/journal.pone.0132301 26196893 PMC4509890

[B17] Derjaguin B. V. Muller V. M. Toporov Y. (1975). Effect of contact deformations on the adhesion of particles. J. Colloid Interface Sci. 53, 314–326. doi: 10.1016/0021-9797(75)90018-1

[B18] Dziuba J. Szerszunowicz I. Nałęcz D. Dziuba M. (2014). Proteomic analysis of albumin and globulin fractions of pea (*Pisum sativum* L.) seeds. Acta Sci. Pol. Technol. Aliment 13, 181–190. doi: 10.17306/j.afs.2014.2.7 24876313

[B19] Eanes E. D. Glenner G. G. (1968). X-Ray diffraction studies on amyloid filaments. J. Histochem. Cytochem. 16, 673–677. doi: 10.1177/16.11.673 5723775

[B20] Fonin A. V. Sulatskaya A. I. Kuznetsova I. M. Turoverov K. K. (2014). Fluorescence of dyes in solutions with high absorbance. Inner filter effect correction. PloS One 9, e103878. doi: 10.1371/journal.pone.0103878 25072376 PMC4114876

[B21] Foreman D. L. Vanderlinde E. M. Bay D. C. Yost C. K. (2010). Characterization of a gene family of outer membrane proteins (ropB) in *Rhizobium leguminosarum* bv. *viciae* VF39SM and the role of the sensor kinase ChvG in their regulation. J. Bacteriol. 192, 975–983. doi: 10.1128/JB.01140-09 20023026 PMC2812955

[B22] Glenn A. R. Poole P. S. Hudman J. F. (1980). Succinate uptake by free-living and bacteroid forms of *Rhizobium leguminosarum* . Microbiol. (N Y) 119, 267–271. doi: 10.1099/00221287-119-1-267

[B23] Gomes V. M. Mosqueda M.-I. Blanco-Labra A. Sales M. P. Fernandes K. V. S. Cordeiro R. A.. (1997). Vicilin storage proteins from *Vigna unguiculata* (legume) seeds inhibit fungal growth. J. Agric. Food Chem. 45, 4110–4115. doi: 10.1021/jf960942g

[B24] Hirsch A. M. (1999). Role of lectins (and rhizobial exopolysaccharides) in legume nodulation. Curr. Opin. Plant Biol. 2, 320–326. doi: 10.1016/S1369-5266(99)80056-9 10458994

[B25] Joseph Sahaya Rajan J. Chinnappan Santiago T. Singaravel R. Ignacimuthu S. (2016). Outer membrane protein C (OmpC) of *Escherichia coli* induces neurodegeneration in mice by acting as an amyloid. Biotechnol. Lett. 38, 689–700. doi: 10.1007/s10529-015-2025-8 26712371

[B26] Kosolapova A. O. Antonets K. S. Belousov M. V. Nizhnikov A. A. (2020). Biological functions of prokaryotic amyloids in interspecies interactions: facts and assumptions. Int. J. Mol. Sci. 21, 7240. doi: 10.3390/ijms21197240 PMC7582709 33008049

[B27] Kosolapova A. O. Belousov M. V. Sulatskaya A. I. Belousova M. E. Sulatsky M. I. Antonets K. S.. (2019). Two novel ayloid proteins, RopA and RopB, from the root nodule bacterium *Rhizobium leguminosarum* . Biomolecules 9, 694. doi: 10.3390/biom9110694 PMC6920782 31690032

[B28] Krol E. Yau H. C. L. Lechner M. Schäper S. Bange G. Vollmer W.. (2020). Tol-pal system and rgs proteins interact to promote unipolar growth and cell division in *Sinorhizobium meliloti* . mBio 11, e00306–20. doi: 10.1128/mBio.00306-20 32605980 PMC7327166

[B29] Kuznetsova I. M. Sulatskaya A. I. Uversky V. N. Turoverov K. K. (2012a). A new trend in the experimental methodology for the analysis of the thioflavin T binding to amyloid fibrils. Mol. Neurobiol. 45, 488–498. doi: 10.1007/s12035-012-8272-y 22592269

[B30] Kuznetsova I. M. Sulatskaya A. I. Uversky V. N. Turoverov K. K. (2012b). Analyzing thioflavin T binding to amyloid fibrils by an equilibrium microdialysis-based technique. PloS One 7, e30724. doi: 10.1371/journal.pone.0030724 22383971 PMC3286464

[B31] Laguerre G. Depret G. Bourion V. Duc G. (2007). *Rhizobium leguminosarum* bv. *viciae* genotypes interact with pea plants in developmental responses of nodules, roots and shoots. New Phytol. 176, 680–690. doi: 10.1111/j.1469-8137.2007.02212.x 17822397

[B32] Laskowska E. Kuczyńska-Wiśnik D. Lipińska B. (2019). Proteomic analysis of protein homeostasis and aggregation. J. Proteomics 198, 98–112. doi: 10.1016/j.jprot.2018.12.003 30529741

[B33] Law I. J. Strijdom B. W. (1984). Role of lectins in the specific recognition of *Rhizobium* by *Lotononis bainesii* . Plant Physiol. 74, 779–785. doi: 10.1104/pp.74.4.779 16663509 PMC1066767

[B34] Levine M. M. Ristaino P. Marley G. Smyth C. Knutton S. Boedeker E.. (1984). Coli surface antigens 1 and 3 of colonization factor antigen II-positive enterotoxigenic *Escherichia coli*: morphology, purification, and immune responses in humans. Infect. Immun. 44, 409–420. doi: 10.1128/iai.44.2.409-420.1984 6370866 PMC263534

[B35] Malovichko Y. V. Shtark O. Y. Vasileva E. N. Nizhnikov A. A. Antonets K. S. (2020). Transcriptomic Insights into Mechanisms of Early Seed Maturation in the Garden Pea (*Pisum sativum* L.). Cells 9, 779. doi: 10.3390/cells9030779 PMC7140803 32210065

[B36] Marquardt D. W. (1963). An algorithm for least-squares estimation of nonlinear parameters. J. Appl. Ind. Math. 11, 431–441. doi: 10.1137/0111030

[B37] Micsonai A. Wien F. Bulyáki É. Kun J. Moussong É. Lee Y.-H.. (2018). BeStSel: a web server for accurate protein secondary structure prediction and fold recognition from the circular dichroism spectra. Nucleic Acids Res. 46, W315–W322. doi: 10.1093/nar/gky497 29893907 PMC6031044

[B38] Montes García J. F. Vaca S. Delgado N. L. Uribe-García A. Vázquez C. Sánchez Alonso P.. (2018). *Mannheimia haemolytica* OmpP2-like is an amyloid-like protein, forms filaments, takes part in cell adhesion and is part of biofilms. Antonie Van Leeuwenhoek 111, 2311–2321. doi: 10.1007/s10482-018-1122-9 29974354

[B39] Morris K. L. Serpell L. C. (2012). “X-Ray fibre diffraction studies of amyloid fibrils,” in Amyloid proteins: Methods and protocols. Eds. Sigurdsson E. M. Calero M. Gasset. M. (Totowa, NJ, USA: Humana Press), 121–135. doi: 10.1007/978-1-61779-551-0_9 22528087

[B40] Nizhnikov A. A. Alexandrov A. I. Ryzhova T. A. Mitkevich O. V. Dergalev A. A. Ter-Avanesyan M. D.. (2014). Proteomic screening for amyloid proteins. PloS One 9, e116003. doi: 10.1371/journal.pone.0116003 25549323 PMC4280166

[B41] Nizhnikov A. A. Antonets K. S. Inge-Vechtomov S. G. (2015). Amyloids: from pathogenesis to function. Biochem. (Mosc.) 80, 1127–1144. doi: 10.1134/S0006297915090047 26555466

[B42] Nizhnikov A. A. Ryzhova T. A. Volkov K. V. Zadorsky S. P. Sopova J. V. Inge-Vechtomov S. G.. (2016). Interaction of prions causes heritable traits in *Saccharomyces cerevisiae* . PloS Genet. 12, e1006504. doi: 10.1371/journal.pgen.1006504 28027291 PMC5189945

[B43] O’Connor D. V. Phillips D. (1984). Time-correlated single photon counting (Cambridge, Massachusetts, USA: Academic Press). doi: 10.1016/B978-0-12-524140-3.X5001-1

[B44] Otzen D. E. Andersen K. K. (2013). Folding of outer membrane proteins. Arch. Biochem. Biophys. 531, 34–43. doi: 10.1016/j.abb.2012.10.008 23131493

[B45] Perez-Riverol Y. Bai J. Bandla C. García-Seisdedos D. Hewapathirana S. Kamatchinathan S.. (2022). The PRIDE database resources in 2022: a hub for mass spectrometry-based proteomics evidences. Nucleic Acids Res. 50, D543–D552. doi: 10.1093/nar/gkab1038 34723319 PMC8728295

[B46] Ren B. Zhang Y. Zhang M. Liu Y. Zhang D. Gong X.. (2019). Fundamentals of cross-seeding of amyloid proteins: an introduction. J. Mater. Chem. B 7, 7267–7282. doi: 10.1039/c9tb01871a 31647489

[B47] Roest H. P. (1995). Outer membrane protein changes during bacteroid development are independent of nitrogen fixation and differ between indeterminate and determinate nodulating host plants of *Rhizobium leguminosarum* . Mol. Plant Microbe Interact. 8, 14. doi: 10.1094/MPMI-8-0014 8589412

[B48] Roest H. P. Mulders I. H. Wijffelman C. A. Lugtenberg B. J. (1995). Isolation of *ropB*, a gene encoding a 22-kDa *Rhizobium leguminosarum* outer membrane protein. Mol. Plant Microbe Interact. 8, 576–583. doi: 10.1094/MPMI-8-0576 8589412

[B49] Romanov V. I. Gordon A. J. Minchin F. R. Witty J. F. Skøt L. James C. L.. (1995). Anatomy, physiology and biochemistry of root nodules of Sprint-2Fix^–^, a symbiotically defective mutant of pea (*Pisum sativum* L.). J. Exp. Bot. 46 (12), 1809–1816. doi: 10.1093/jxb/46.12.1809

[B50] Romero D. Kolter R. (2014). Functional amyloids in bacteria. Int. Microbiol. 17, 65–73. doi: 10.2436/20.1501.01.208 26418850

[B51] Rose T. L. Gomes V. M. da Cunha M. Fernandes K. V. S. Xavier-Filho J. (2003). Effect of sugars on the association between cowpea vicilin (7S storage proteins) and fungal cells. Biocell 27, 173–179. doi: 10.32604/biocell.2003.27.173 14510235

[B52] Rosov F. N. Shleev S. V. Petrova N. E. Tsyganov V. E. Borisov A. Y. Topunov A. F.. (2001). The *Sym31* gene responsible for bacteroid differentiation is involved in nitrate-dependent nodule formation in pea plants. Russ. J. Plant Physiol. 48(4), 459–463. doi: 10.1023/a:1016743111625

[B53] Sønderby T. V. Najarzadeh Z. Otzen D. E. (2022). Functional bacterial amyloids: Understanding fibrillation, regulating biofilm fibril formation and organizing surface assemblies. Molecules 27, 4080. doi: 10.3390/molecules27134080 35807329 PMC9268375

[B54] Sacher J. C. Shajahan A. Butcher J. Patry R. T. Flint A. Hendrixson D. R.. (2020). Binding of phage-encoded FlaGrab to motile *Campylobacter jejuni* flagella inhibits growth, downregulates energy metabolism, and requires specific flagellar glycans. Front. Microbiol. 11. doi: 10.3389/fmicb.2020.00397 PMC7099621 32265863

[B55] Sambrook J. Fritsch E. F. Maniatis T. (1989). “Molecular cloning,” in A laboratory manual, 2nd ed (New York, NY, USA: Cold Spring Harbor Laboratory Press: Cold Spring Harbour).

[B56] Sawaya M. R. Hughes M. P. Rodriguez J. A. Riek R. Eisenberg D. S. (2021). The expanding amyloid family: Structure, stability, function, and pathogenesis. Cell 184, 4857–4873. doi: 10.1016/j.cell.2021.08.013 34534463 PMC8772536

[B57] Serio T. R. Cashikar A. G. Moslehi J. J. Kowal A. S. Lindquist S. L. (1999). “Yeast prion [Ψ+] and its determinant, sup35p,” in Methods in enzymology, vol. 649–673 (The Netherlands: Elsevier B.V.). doi: 10.1016/S0076-6879(99)09043-6 10507053

[B58] Sherrier D. J. Borisov A. Y. Tikhonovich I. A. Brewin N. J. (1997). Immunocytological evidence for abnormal symbiosome development in nodules of the pea mutant line Sprint-2Fix^–^ (*sym31*). Protoplasma 199(1), 57–68. doi: 10.1007/bf02539806

[B59] Shutov A. D. Kakhovskaya I. A. Braun H. Bäumlein H. Müntz K. (1995). Legumin-like and vicilin-like seed storage proteins: evidence for a common single-domain ancestral gene. J. Mol. Evol. 41, 1057–1069. doi: 10.1007/BF00173187 8587104

[B60] Soudy M. Anwar A. M. Ahmed E. A. Osama A. Ezzeldin S. Mahgoub S.. (2020). UniprotR: Retrieving and visualizing protein sequence and functional information from universal protein resource (UniProt knowledgebase). J. Proteomics 213, 103613. doi: 10.1016/j.jprot.2019.103613 31843688

[B61] Studier F. W. Moffatt B. A. (1986). Use of bacteriophage T7 RNA polymerase to direct selective high-level expression of cloned genes. J. Mol. Biol. 189, 113–130. doi: 10.1016/0022-2836(86)90385-2 3537305

[B62] Subedi S. Sasidharan S. Nag N. Saudagar P. Tripathi T. (2022). Amyloid cross-seeding: Mechanism, implication, and inhibition. Molecules 27, 1776. doi: 10.3390/molecules27061776 35335141 PMC8955620

[B63] Sulatskaya A. I. Kosolapova A. O. Bobylev A. G. Belousov M. V. Antonets K. S. Sulatsky M. I.. (2021). β-barrels and amyloids: Structural transitions, biological functions, and pathogenesis. Int. J. Mol. Sci. 22, 11316. doi: 10.3390/ijms222111316 34768745 PMC8582884

[B64] Sunde M. Blake C. (1997). “The structure of amyloid fibrils by electron microscopy and X-ray diffraction,” in Advances in protein chemistry, vol. 50. (The Netherlands: Elsevier B.V.), 123–159. doi: 10.1016/S0065-3233(08)60320-4 9338080

[B65] Tolin S. Arrigoni G. Moscatiello R. Masi A. Navazio L. Sablok G.. (2013). Quantitative analysis of the naringenin-inducible proteome in *Rhizobium leguminosarum* by isobaric tagging and mass spectrometry. Proteomics 13, 1961–1972. doi: 10.1002/pmic.201200472 23580418

[B66] Tsyganova A. V. Seliverstova E. V. Brewin N. J. Tsyganov V. E. (2019). Comparative analysis of remodelling of the plant-microbe interface in *Pisum sativum* and *Medicago truncatula* symbiotic nodules. Protoplasma 256, 983–996. doi: 10.1007/s00709-019-01355-5 30793221

[B67] Tsyganov V. E. Tsyganova A. V. (2020). Symbiotic regulatory genes controlling nodule development in *Pisum sativum* L. Plants 9, 1741. doi: 10.3390/plants9121741 PMC7764586 33317178

[B68] Tsyganov V. E. Voroshilova V. A. Herrera-Cervera J. A. Sanjuan-Pinilla J. M. Borisov A. Y. Tikhonovich I. A.. (2003). Developmental downregulation of rhizobial genes as a function of symbiosome differentiation in symbiotic root nodules of *Pisum sativum* . New Phytol. 159(2), 521–530. doi: 10.1046/j.1469-8137.2003.00823.x 33873360

[B69] van Gerven N. van der Verren S. E. Reiter D. M. Remaut H. (2018). The role of functional amyloids in bacterial virulence. J. Mol. Biol. 430, 3657–3684. doi: 10.1016/j.jmb.2018.07.010 30009771 PMC6173799

[B70] Vedam V. Kannenberg E. Datta A. Brown D. Haynes-Gann J. G. Sherrier D. J.. (2006). The pea nodule environment restores the ability of a *Rhizobium leguminosarum* lipopolysaccharide *acpXL* mutant to add 27-hydroxyoctacosanoic acid to its lipid A. J. Bacteriol. 188, 2126–2133. doi: 10.1128/JB.188.6.2126-2133.2006 16513742 PMC1428142

[B71] Vladimirov Y. A. Litvin F. F. (1964). “Photobiology and spectroscopic methods,” in Handbook of general biophisics (Moscow, Russia: High school), 88–91.

[B72] Wang F. Wang Y. Jiang L. Wang W. Sang J. Wang X.. (2021). The food additive fast green FCF inhibits α-synuclein aggregation, disassembles mature fibrils and protects against amyloid-induced neurotoxicity. Food Funct. 12, 5465–5477. doi: 10.1039/D0FO03301D 33997868

[B73] Wheatley R. M. Ford B. L. Li L. Aroney S. T. N. Knights H. E. Ledermann R.. (2020). Lifestyle adaptations of *Rhizobium* from rhizosphere to symbiosis. Proc. Natl. Acad. Sci. 117, 23823–23834. doi: 10.1073/pnas.2009094117 32900931 PMC7519234

[B74] Yang J. Lan L. Jin Y. Yu N. Wang D. Wang E. (2022). Mechanisms underlying legume-rhizobium symbioses. J. Integr. Plant Biol. 64, 244–267. doi: 10.1111/jipb.13207 34962095

[B75] Yao Z. Xie Y. Wang L. Yan C. Du H. Hu H.. (2022). A comparative study of indirect calorimetry and prediction equations in overweight and obese Chinese adults. Nutr. Clin. Metab 36, 190–196. doi: 10.1016/j.nupar.2022.04.001

[B76] Young J. P. W. Moeskjær S. Afonin A. Rahi P. Maluk M. James E. K.. (2021). Defining the *Rhizobium leguminosarum* species complex. Genes 12, 111. doi: 10.3390/genes12010111 33477547 PMC7831135

