# Supplementary material for: RopB protein of Rhizobium leguminosarum bv. viciae adopts amyloid state during symbiotic interactions with pea (Pisum sativum L.)
Source: Front Plant Sci. 2022 Oct 24;13:1014699. doi: 10.3389/fpls.2022.1014699 (PMC9650718; doi:10.3389/fpls.2022.1014699)
Supplement: Supplementary file 1 [file DataSheet_1.pdf]

Supplementary Material

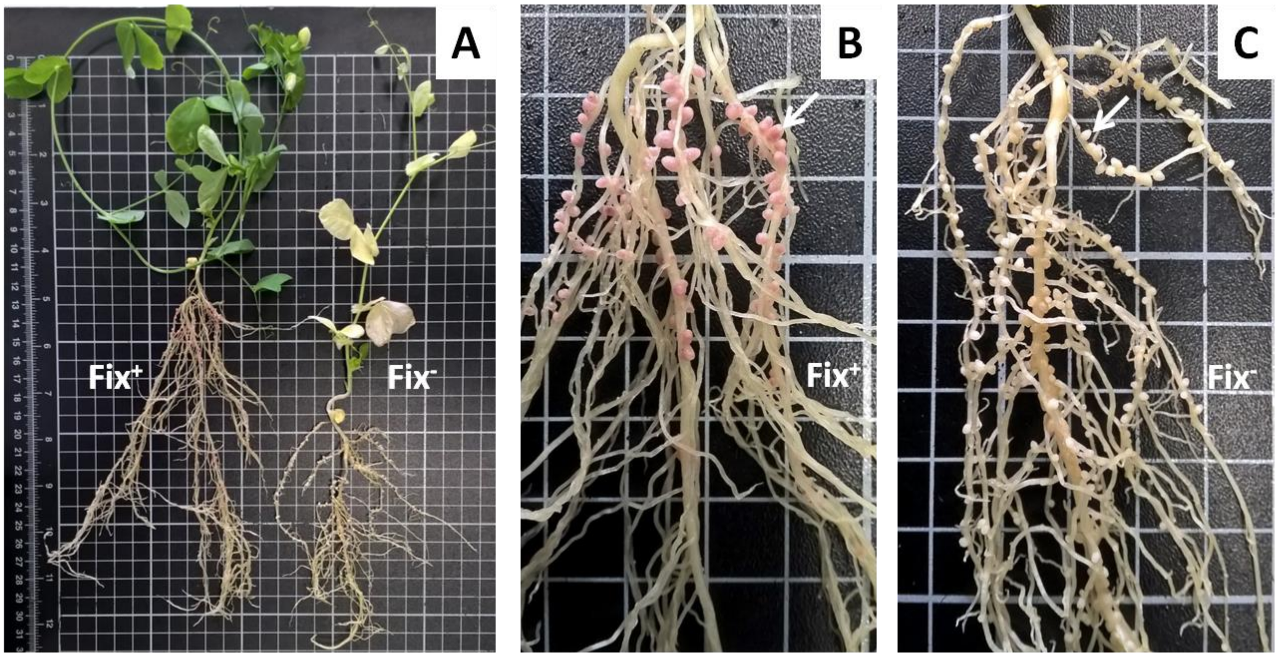

Supplementary Figure 1. Development of plants of *Pisum sativum* L. lines Sprint-2 (Fix$^+$ phenotype) and Sprint-2Fix$^-$ (*sym31*; Fix$^-$ phenotype) inoculated with *Rhizobium leguminosarum* bv. *viciae* strain RCAM 1026 (25 days after inoculation). **(A)** Appearance of the whole plants. **(B, C)** Sprint-2 **(B)** and Sprint-2Fix$^-$ **(C)** root systems with nodules (arrowed). One grid division is 10 mm.

Supplementary Table 1. Biometric parameters of the wild-type *Pisum sativum* L. plants Sprint-2 and the mutant Sprint-2Fix$^-$ (*sym31*) 25 days after inoculation with *Rhizobium leguminosarum* bv. *viciae* RCAM1026.

Plant genotype	Whole plant fresh weight, g (n = 15)	Shoot fresh weight, g (n = 15)	Nodule number per plant, pcs. (n = 3)	Nodule fresh weight per plant, g (n = 3)	Fresh weight of one nodule, mg
Sprint-2 (Wt)	4.283	1.992	94.7	0.093	1.037
Sprint-2Fix$^-$ (*sym31*)	1.636*	0.775*	215.3*	0.133	0.623

* – the values of the mutant and the original line differ significantly ($p \leq 0.05$). Pairwise comparison of data was performed using the One-Way ANOVA test in the SigmaPlot 12.0 program (SPSS Inc Chicago, IL, USA).

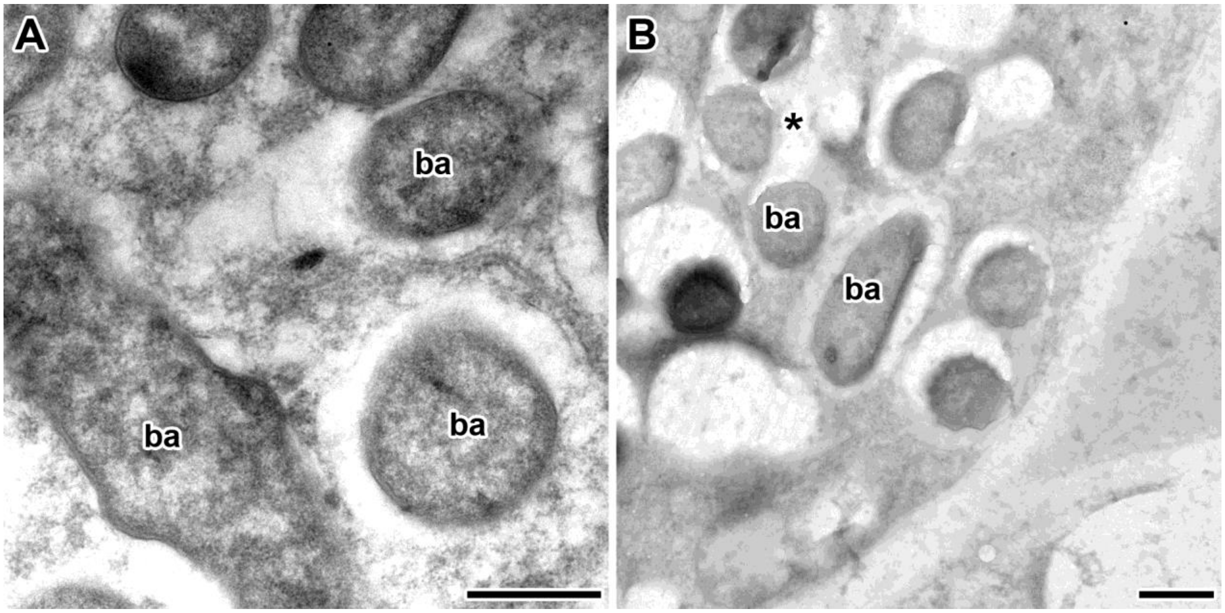

Supplementary Figure 2. Transmission electron micrographs of cells from *Pisum sativum* L. nodule treated as negative control. Gold particles were absent when cells were treated with unspecific secondary antibody **(A)**, after the omission of the primary antibody **(B)**. ba – bacteroid, * – multibacteroid symbiosome. (**A)** Wild-type line Sprint-2 of *Pisum sativum* L. labelled with RopB antibody and treated with anti-mouse secondary antibody conjugated to 10 nm diameter colloidal gold. (**B)** Mutant line Sprint-2Fix^{-} (*sym31*) of *Pisum sativum* L. after omission of the primary antibody. Bar: **(A)** = 200 nm, **(B)** = 500 nm.

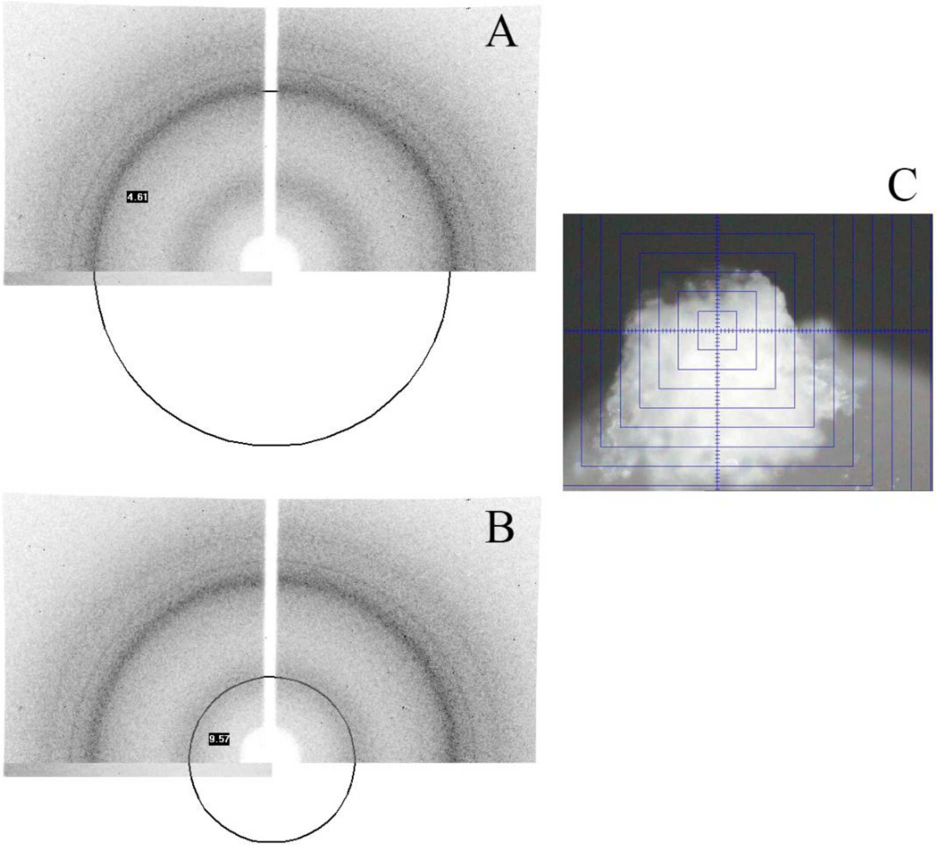

Supplementary Figure 3. (A) and **(B)** x-ray diffraction analysis revealed ring-shaped x-ray reflections of RopB fibrils. Calibration rings corresponding to distances of 9.57 Å and 4.61 Å are shown on top of the reflections. **(C)** A crystal of aggregated RopB on the surface of a paraffin head prepared according to the technique described in Materials and Methods (x-ray diffraction analysis) of the manuscript. The x-ray beam pointing area is shown.

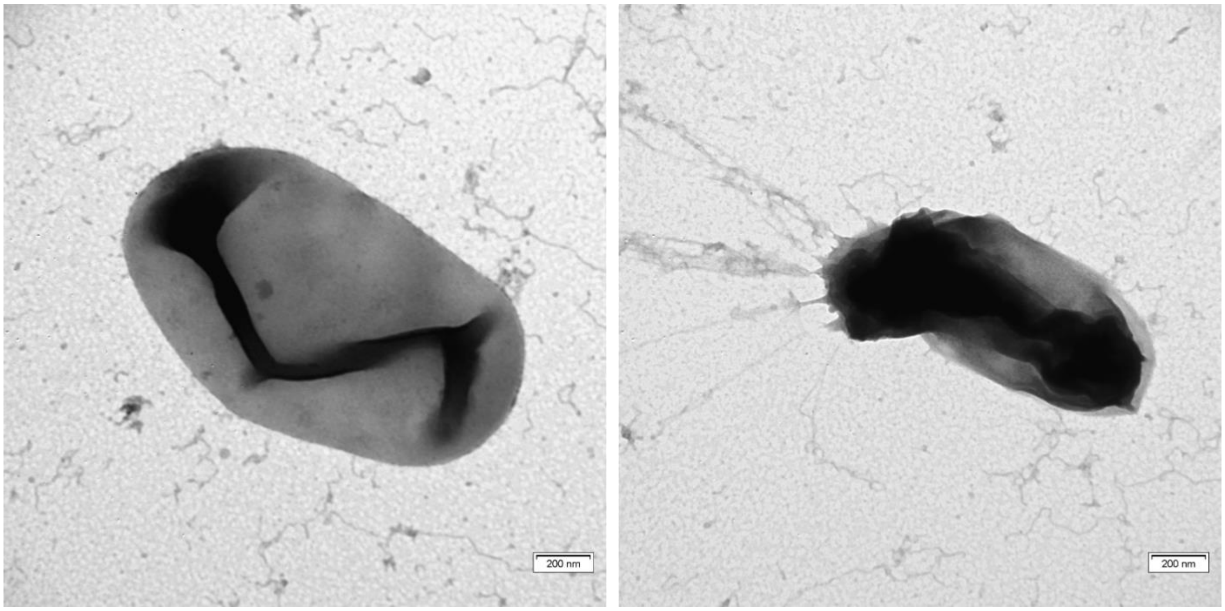

Supplementary Figure 4. Transmission electron micrographs of bacteroids treated as negative control. Bacteroids from mutant line Sprint-2Fix^{-} (*sym31*) of *Pisum sativum* L. treated only with anti-rabbit secondary antibody conjugated to 10 nm diameter colloidal gold. Bar = 200 nm.

1 MRVLIAGLMA SVFAIAGVSA AQAADRVDQV PEAPVAQEAP VKPAGSWEGF YLGGAGTYNM GDFGSDRHTY GFGGQVFTGY

81 NWQQGQIVYG VESDLGYSGD DVSSGGVENK YGWNGSVRGR **VGYDMNPFLL YGTAGLAIGD VK**VSDDTSDE SK**TNFGYTVG**

161 **AGVEAFVTNN ITTR**LEYR**YT DYQSKDYDLD SGSFSR**GYDE NSVKLGIGVK F

Supplementary Figure 5. Coverage of RopB protein with peptides identified with mass-spectrometry. Amino acid sequence of RopB is shown. Peptides identified by mass spectrometry are highlighted and underlined.

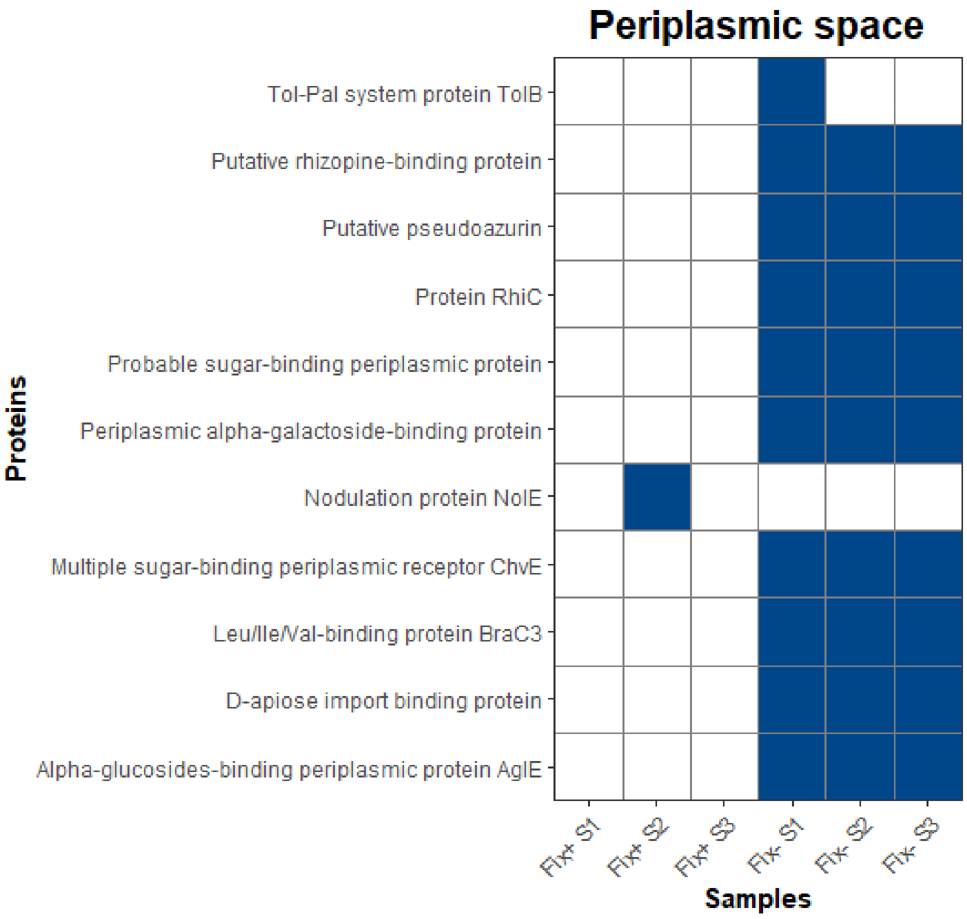

Supplementary Figure 6. Potentially amyloidogenic proteins of *Rhizobium leguminosarum* with "periplasmic space" cellular component GO term identified in protein samples obtained from Fix$^+$ and Fix$^-$ nodules. Proteins identified in particular samples (S1-S3) and Fix status of nodules are shown.

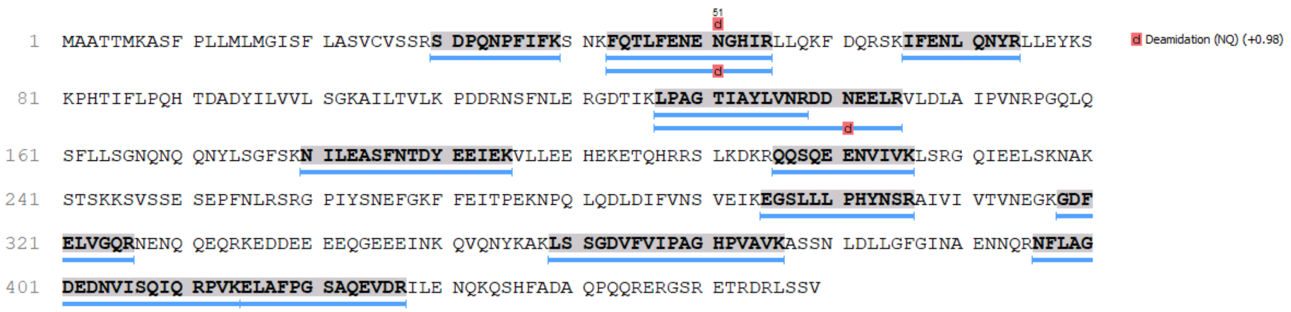

Supplementary Figure 7. Coverage of vicilin protein with peptide identified with mass-spectrometry. Amino acid sequence of vicilin is shown. Peptides identified by mass spectrometry are highlighted and underlined. Amino acid modifications are marked.
